# Molecular biology of the deadliest cancer – glioblastoma: what do we know?

**DOI:** 10.3389/fimmu.2025.1530305

**Published:** 2025-03-21

**Authors:** Aly Ismailov, Aldo Spallone, Alexey Belogurov, Alan Herbert, Maria Poptsova

**Affiliations:** ^1^ International Laboratory of Bioinformatics, Institute of Artificial Intelligence and Digital Sciences, Faculty of Computer Science, National Research University Higher School of Economics, Moscow, Russia; ^2^ Laboratory of Hormonal Regulation Proteins, Shemyakin and Ovchinnikov Institute of Bioorganic Chemistry Russian Academy of Sciences (RAS), Moscow, Russia; ^3^ Scientific and Educational Institute of Fundamental Medicine named after V.I. Pokrovsky, Department of Biological Chemistry, Russian University of Medicine, Moscow, Russia; ^4^ Discovery Department, InsideOutBio, Boston, MA, United States

**Keywords:** glioblastoma, glioma, ligand-receptor interaction, signaling pathways, tumor microenvironment, glioblastoma stem cells, cancer-associated fibroblasts, immunotherapy

## Abstract

Glioblastomas are the most prevalent primary brain tumors and are associated with a dramatically poor prognosis. Despite an intensive treatment approach, including maximal surgical tumor removal followed by radio- and chemotherapy, the median survival for glioblastoma patients has remained around 18 months for decades. Glioblastoma is distinguished by its highly complex mechanisms of immune evasion and pronounced heterogeneity. This variability is apparent both within the tumor itself, which can exhibit multiple phenotypes simultaneously, and in its surrounding microenvironment. Another key feature of glioblastoma is its “cold” microenvironment, characterized by robust immunosuppression. Recent advances in single-cell RNA sequencing have uncovered new promising insights, revealing previously unrecognized aspects of this tumor. In this review, we consolidate current knowledge on glioblastoma cells and its microenvironment, with an emphasis on their biological properties and unique patterns of molecular communication through signaling pathways. The evidence underscores the critical need for personalized poly-immunotherapy and other approaches to overcome the plasticity of glioblastoma stem cells. Analyzing the tumor microenvironment of individual patients using single-cell transcriptomics and implementing a customized immunotherapeutic strategy could potentially improve survival outcomes for those facing this formidable disease.

## 1 Introduction

Glioblastomas (GBM) are the most common primary brain tumors, characterized by extremely poor prognosis. Approximately 16% of all primary central nervous system neoplasms are glioblastomas (1). Despite the aggressive treatment strategy, which includes gross total surgical tumor resection followed by courses of radio- and chemotherapy, the survival rate for glioblastoma patients has remained at approximately 18 months for years (2).

**Figure 1:**
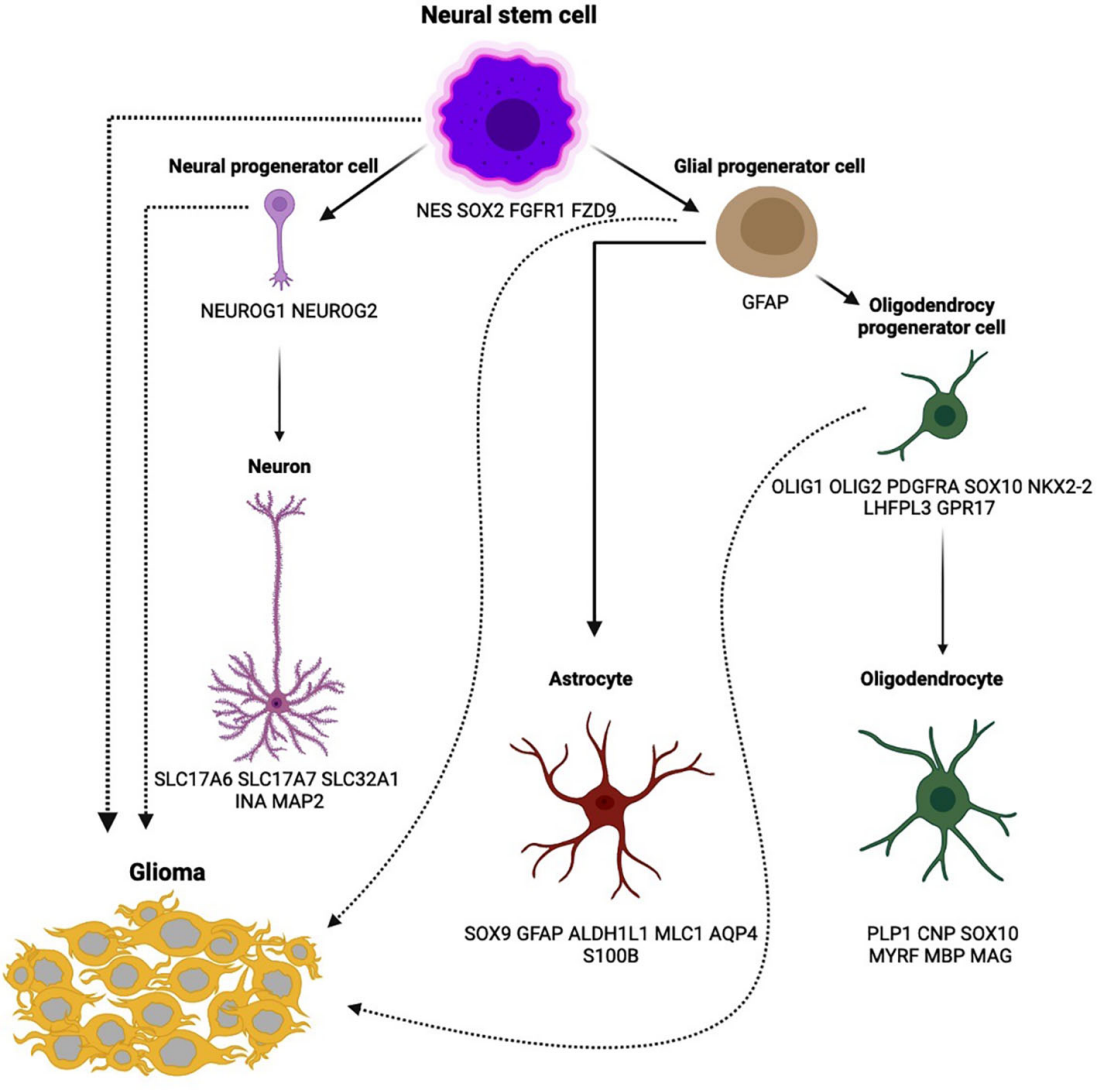
Cell types of neural tissue with corresponding markers that can be transformed to glioma.

According to the 2021 WHO classification of central nervous system tumors, a diffuse IDH-wildtype astrocytoma can be classified as a glioblastoma if it meets any one of the following five criteria: microvascular proliferation, necrosis, *TERT* promoter mutation, *EGFR* gene amplification, or chromosome copy number alterations (+7/–10) (3). Glioblastoma cells are believed to originate from neural stem cells (NSCs), which are progenitors for neuronal, astrocytic, and oligodendrocytic lineages (4). NSCs possess self-renewal and proliferative potential and are primarily located in the subventricular regions of the brain. These cells can migrate to other brain areas, serving as a substrate for the development of malignant gliomas (5, 6). Cell types of neural tissue with corresponding markers that can be transformed to glioma (7–9) are presented in **Figure 1**. The diverse glioma morphologies reflect the highly plastic and proliferative nature of normal glial development. The inherent adaptability that characterizes a glioblastoma stem cell makes these cancers a challenge to cure.

## 2 Glioblastoma heterogeneity

**Figure 2:**
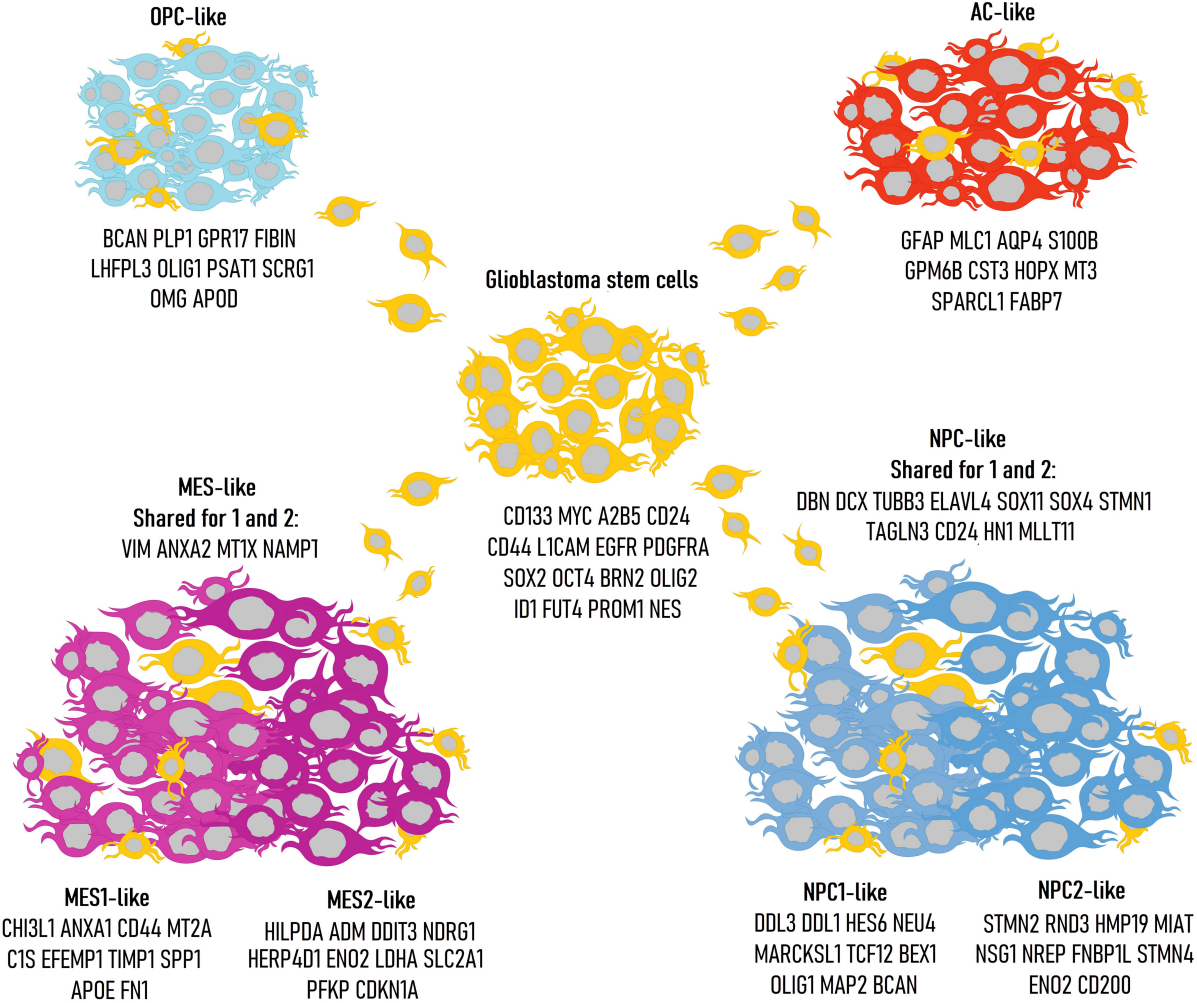
Classification of glioblastoma cell types with corresponding genetic markers: glioblastoma stem cells (GSCs), AC-like, MES-like, NPC-like, OPC-like. The different presentations highlight the inherent plasticity of glioblastoma stem cells.

A key component of the aggressive biology of glioblastoma is the extreme heterogeneity harboring in its genetic profile. In 2010, Verhaak et al. used bulk sequencing to identify four clinically significant glioblastoma subtypes: classical, mesenchymal, proneural, and neural. These classifications were based on specific genetic mutations: *EGFR*, *NF1*, and *PDGFRA/IDH1/TP53*, respectively, with the neural subtype exhibiting expression of standard neuronal markers such as *NEFL*, *GABRA1*, *SYT1*, and *SLC12A5* (10). The proneural subtype often corresponds to secondary glioblastoma (as classified in the 2021 WHO CNS tumor guidelines as astrocytoma grade 4), which arises from the anaplastic transformation of less malignant glioma forms. With the advent of single-cell sequencing, this classification has been refined, now identifying mesenchymal-like (MES-like), astrocyte-like (AC-like), oligodendrocyte progenitor-like (OPC-like), and neural progenitor-like (NPC-like) types (11). Each of these cellular types has distinct markers indicating mesenchymal, astrocytic, oligodendroglial, or neuronal progenitor gene expression (**Figure 2**). Additionally, there exists a range of stromal markers expressed by these glioblastoma types (**Table 1**).

**Table 1:** Gene markers for cell types and subtypes.ne markers for cell types/subtypes.

Cell type/subtype	Gene markers
**AC-like**	*GFAP*, *MLC1*, *AQP4*, *S100B*, *GPM6B, TSPAN7*, *PTPRZ1*, *HEPN1*, *NDRG2*, *CLU, HOPX, RAMP1*, *EDNRB*, *AGT, PON2*, *S100A16*, *CST3*, *PLTP*, *PPAP2B*, *RAB31*, *DBI*, *PMP2*, *GATM*, *SPARC*, *SPARCL1*, *TTYH1*, *ATP1A2*, *ATP1B2*, *PMP22, F3*, *ANXA5*, MT3, *METTL7B.*
**OPC-like**	*PLLP*, *PLP1*, *CNP*, *OMG*, *BCAS1*, *SIRT2*, *PMP2*, *GPM6B*, *RTKN*, *P2RX7*, *CADM2, PSAT1, LHFPL3*, *GPR17*, *OLIG1*, *NKAIN4*, *THY1*, *FGF12*, *DBI*, *LPPR1*, *RAB31*, *NLGN3*, *NEU4*, *HRASLS*, *SERINC5*, *SCRG1*, *TNS3*, *GPR37L1*, *BCAN*, *VCAN*, *PTPRZ1*, *CNTN1, FIBIN, APOD, TTYH1*, *TMEM206*, *FXYD6, RNF13*, *EPB41L2*, *ALCAM*, *PCDHGC3*, *PGRMC1.*
**MES1 and MES2**	*VIM, ANXA2, LGALS3, MT1X, NAMPT.*
**1. MES1-like**	*ANXA1, CHI3L1, IFITM3, C1S, C1R, C3, SERPING1, TNFRSF1A, SERPINA3, MGST1, CD44, FN1, SPP1, LGALS1, LGALS3, SOD2, PRDX6, MT2A, TIMP1, IGFBP7, WWTR1, S100A10, S100A11.*
**2. MES2-like**	*HILPDA, DDIT3, NDRG1, HERPUD1, TRIB3, EPAS1, EGLN3, BNIP3, BNIP3L, GDF15, NAMPT, ADM, ENO2, SLC2A1, SLC2A3, PFKP, PGK1, LDHA, CDKN1A, IGFBP3.*
**NPC1 and NPC2**	*DBN1*, *DCX*, *ELAVL4*, *SOX11*, *STMN1*, *TAGLN3*, *TUBB3, CD24*, *HN1*, *MLLT11*, *SOX4.*
**1. NPC1-like**	*ASCL1*, *HES6*, *OLIG1*, *MYT1*, *TCF12*, *BEX1*, *CHD7*, *GPR56*, *DLL1*, *DLL3*, *ETV1*, *TSPAN13*, *PCBP4*, *SEZ6L*, *SEZ6*, *MAP2*, *NEU4*, *HIP1, MARCKSL1, BEX1, GRIK2*, *ABAT, TNR*, *NXPH1.*
**2. NPC2-like**	*MAP1B*, *TUBB2A*, *DPYSL3*, *DPYSL5*, *STMN2*, *STMN4*, *FNBP1L*, *PAK3*, *RND3*, *MIAT*, *KIF5C*, *KIF5A*, *DYNC1I1, TCF4*, *RBFOX2*, *NFIB*, HMP19, NREP, NSG1, *SNAP25*, *ENO2*, *SEPT3*, *CD200.*
**GSCs**	*CD133, MYC, A2B5, CD24, CD44, L1CAM, EGFR, PDGFRA, SOX2, OCT4, BRN2, OLIG2, ID1, FUT4, PROM1, NES.*
**Macrophages**	*F10, EMILIN2, F5, C3, GDA, MKI67, SELL, HP, ICAM1, CCR2, C1QA, CD207, CD209.*
**Microglia**	*P2RY13, P2RY12, GPR34, SLC2A5, OLFML3, TMEM119, FCRLA, SALL1, TREM2.*
**All T-cells**	*CD3, TCR.*
**1. T-killer cells (CTLs)**	*CD8, PRF1, GZMB, GZMA, GZMH, NKG7, GNLY.*
**2. All T-helper cells** **- T-helper 1** **- T-helper 2** **- T-helper 17**	*CD4, CCR5, CXCR4.*
	*TNF-α.*
	*IL-4, IL-5, IL-13.*
	*T17, IL-17A, IL-17F, IL-22.*
**3. T-regulatory cells**	*CD4, CD25, FOXP3, CTLA4.*
**B-cells**	*CD45, CD19, CD20.*
**NK-cells**	*NCR1, KLRD1, FCGR3A, KLRC1, KLRC3, KLRB1, KLRC2, NCR3, NCR2.*
**MSCs**	*CD90, CD105, CD73.*
**CAFs**	*ACTA2, FAP, PDGFRA, PDGFRB, PDPN, S100A4, TNC, VIM, COL1A1, CCDC80, BGN, COL4A2, COL5A1, NR2F2, COL3A1, INHBA, STC2, LOXL2, ACTA2, COL1A1, TNC.*

### 2.1 Mesenchymal-like glioblastoma cells

The MES-like type is divided into two subgroups: MES1-like and MES2-like (11). Both subgroups are characterized by the expression of the mesenchymal gene *VIM*, which encodes vimentins, a class III intermediate filament protein. Both subgroups also exhibit activity of *ANXA2*, *LGALS3*, *MT1X*, and *NAMPT*. The MES1-like subgroup shows activity in genes involved in immune and inflammatory responses (*ANXA1*, *CHI3L1*, *IFITM3*, *C1S*, *C1R*, *C3*, *SERPING1*, *TNFRSF1A*, *SERPINA3*, *MGST1*), cellular adhesion, interactions with the extracellular matrix (*CD44*, *FN1*, *SPP1*, *LGALS1*, *LGALS3*), antioxidant activity (*SOD2*, *PRDX6*, *MT2A*), and cell proliferation (*TIMP1*, *IGFBP7*, *WWTR1*, *S100A10*, *S100A11*). The MES2-like subgroup, on the other hand, is associated with the expression of genes linked to hypoxia adaptation, stress responses, and apoptosis (*HILPDA*, *DDIT3*, *NDRG1*, *HERPUD1*, *TRIB3*, *EPAS1*, *EGLN3*, *BNIP3*, *BNIP3L*, *GDF15*, *NAMPT*, *ADM*), glucose metabolism (*ENO2*, *SLC2A1*, *SLC2A3*, *PFKP*, *PGK1*, *LDHA*), and cell proliferation (*CDKN1A*, *IGFBP3*). Thus, the MES2-like state, in some tumors, may be linked to hypoxia and high glucose consumption. MES1-like has been identified as a hypoxia-independent subtype, while MES2-like is considered hypoxia-dependent.

### 2.2 Astrocyte-like glioblastoma cells

The AC-like subgroup expresses markers typical of astrocytic glia (*GFAP*, *MLC1*, *AQP4*, *S100B*, *GPM6B*) (11). This group is also characterized by the expression of genes associated with apoptosis, cell growth, and differentiation (*TSPAN7*, *PTPRZ1*, *HEPN1*, *NDRG2*, *CLU, HOPX*), signaling molecules (*RAMP1*, *EDNRB*, *AGT*), antioxidant activity and cellular metabolism (*PON2*, *S100A16*, *CST3*, *PLTP*, *PPAP2B*, *RAB31*, *DBI*, *PMP2*, *GATM*, *SPARC*, *SPARCL1*, *TTYH1*, *ATP1A2*, *ATP1B2*, *PMP22*), coagulation (*F3*, *ANXA5*), and metal-binding protein (*MT3*). Additionally, *METTL7B*, a gene associated with glioblastoma cell growth and survival, is expressed in this group (12).

### 2.3 Oligodendrocyte-progenitor-like glioblastoma cells

The OPC-like group is characterized by broad expression of two groups of genes: the first group is involved in myelination processes and is actively expressed in oligodendrocytes (*PLLP*, *PLP1*, *CNP*, *OMG*, *BCAS1*, *SIRT2*, *PMP2*, *GPM6B*, *RTKN*, *P2RX7*, *CADM2, PSAT1*), while the second group is predominantly expressed in oligodendrocyte progenitors (*LHFPL3*, *GPR17*, *OLIG1*, *NKAIN4*, *THY1*, *FGF12*, *DBI*, *LPPR1*, *RAB31*, *NLGN3*, *NEU4*, *HRASLS*, *SERINC5*, *SCRG1*, *TNS3*, *GPR37L1*, *BCAN*, *VCAN*, *PTPRZ1*, *CNTN1, FIBIN, APOD*) (11). Additionally, the OPC-like group may express genes related to ion channels (*TTYH1*, *TMEM206*, *FXYD6*), cell proliferation and division (*RNF13*, *EPB41L2*), and adhesion and intercellular interactions (*ALCAM*, *PCDHGC3*, *PGRMC1*).

### 2.4 Neural progenitor-like glioblastoma cells

Like the MES-like group, the NPC-like group is also divided into two subgroups: NPC1-like and NPC2-like (11). Both subgroups are characterized by the expression of genes involved in neurogenesis processes (*DBN1*, *DCX*, *ELAVL4*, *SOX11*, *STMN1*, *TAGLN3*, *TUBB3*), as well as cell differentiation and proliferation (*CD24*, *HN1*, *MLLT11*, *SOX4*). In addition to the activity of common genes for both NPC1 and NPC2 that are active in neural development, each group also expresses a specific set of genes. For the NPC1-like subgroup, the characteristic expression includes genes such as *ASCL1*, *HES6*, *OLIG1*, *MYT1*, *TCF12*, *BEX1*, *CHD7*, *GPR56*, *DLL1*, *DLL3*, *ETV1*, *TSPAN13*, *PCBP4*, *SEZ6L*, *SEZ6*, *MAP2*, *NEU4*, *HIP1, MARCKSL1, BEX1*. Notably, there is also expression of the glutamate kainate receptor and the enzyme involved in the utilization of gamma-aminobutyric acid (*GRIK2*, *ABAT*), as well as neural-specific adhesion proteins (*TNR*, *NXPH1*), which are typical of neurons in the brain. In the case of NPC2-like, the expression is characterized by a broad spectrum of genes crucial for neural development, encoding cytoskeletal proteins and motor proteins: *MAP1B*, *TUBB2A*, *DPYSL3*, *DPYSL5*, *STMN2*, *STMN4*, *FNBP1L*, *PAK3*, *RND3*, *MIAT*, *KIF5C*, *KIF5A*, *DYNC1I1*. Genes specific to this subgroup, which are involved in neural system development, include *TCF4*, *RBFOX2*, *NFIB*, as well as genes active in mature neurons such as *HMP19, NREP, NSG1, SNAP25*, *ENO2*, *SEPT3*, and *CD200*.

### 2.5 Bulk versus single-cell RNA-seq classification

Opposite to the MES-like subtype, the other subtypes are characterized by the expression of genes related either to neurons/neuronal glia or their precursor cells. These subtypes can coexist within a single tumor and, importantly, can transit into one another during biochemical reprogramming (stress, hypoxia) (13). Thus, the previously discussed bulk classification shows strong consistency with the new single-cell classification. The classical and mesenchymal subtypes align well with the AC-like and MES-like subtypes, respectively. The proneural subtype corresponds to a combination of two cellular states: NPC-like and OPC-like, reflecting their typical coexistence within the same tumor. According to observations, the AC-like phenotype in half of the cases undergoes transition to MES-like during disease progression. In the MES-like state, there is a high infiltration of stromal and myeloid cells, which may contribute to the more malignant course of the disease in individuals with this phenotype (14). The previously described neural type likely consists largely of oligodendrocytes and neurons rather than malignant cells. As with the classical type, according to the bulk classification, the AC-like phenotype is characterized by the overexpression of *EGFR*. This is likely due to EGFR’s involvement in astrocyte differentiation, and thus, the overexpression of this gene may secondarily contribute to the manifestation of the AC-like phenotype (14). Additionally, *PDGFRA* and *CDK4* are associated with OPC-like and NPC-like phenotypes, respectively, which is not surprising given the important roles of these genes in oligodendrocyte and neuron development (15, 16). *NF1*, expressed in the mesenchymal type according to the bulk classification, is also characteristic of the MES-like phenotype.

Another characteristic of the tumor under consideration is its heterogeneity in terms of the various subtypes within a single tumor and the differing copy numbers of genes such as *EGFR*, *PTEN*, and *PDGFR*. An increase in the number of copies of these genes is negatively correlated with patients’ survival rate (17, 18). This heterogeneity partly explains the disappointing results of the Rindopepimut trial, a cancer vaccine targeting EGFR-mutant glioblastoma cells (19).

### 2.6 Glioblastoma stem cells

In several malignant neoplasms, subpopulations of cells with stem cell properties have been identified, such as self-renewal capacity, oncogenic potential, and expression of embryonic or tissue-specific stem cell genes. These cells are referred to as cancer stem cells (CSC). In glioblastoma, such subpopulations are called glioblastoma stem cells (GSCs). GSCs exhibit significant resistance to radio- and chemotherapy, and their interaction with the tumor microenvironment profoundly impacts genetic reprogramming and immune resistance (20, 21). Markers of GSCs include *CD133, MYC, A2B5, CD24, CD44, L1CAM, EGFR, PDGFRA, SOX2, OCT4, BRN2, OLIG2, ID1, FUT4, PROM1*, and *NES* (21–26). It has been shown that each of the four types (OPC, NPC, AC, and MES) exhibits significant deviations in the expression of specific GSC markers. Thus, *CD24* is characteristic of NPC-like GSCs, *CD133* of OPC-like, *EGFR* and *NES* of AC-like, and *CD44* of MES-like. The expression of other markers was either characteristic of two types or lacked specificity altogether. Since GSCs represent a small subpopulation of the entire tumor, their identification is challenging (27, 28). The role and functions of GSCs in glioblastoma are poorly understood and remain an area of particular interest for researchers.

## 3 Glioblastoma microenvironment

Another critical aspect of oncogenesis is the tumor microenvironment (TME). The biological characteristics and structural features of the TME are closely related to processes such as oncogenesis, invasion, metastasis, and even pharmacological resistance to certain chemotherapeutic agents (29, 30). It is proposed that the TME may play a dual role in either actively promoting or inhibiting tumor development, largely depending on the complex biochemical cascades influenced by the tumor (31). A focused investigation into the interactions between the tumor and its microenvironment may provide greater insights into tumor biology and reveal new therapeutic strategies, particularly those based on immunotherapeutic approaches (4). The glioblastoma microenvironment can be divided into three distinct regions: the hypoxic niche in the center, the perivascular niche, and the vascular-invasive niche, each characterized by different cellular populations (32). In addition to neurons, astrocytes, and oligodendrocytes, a significant and crucial portion of the non-neoplastic cells in the TME consists of immune elements. The primary components of the CD45^+^ (a general leukocyte marker) immune population in glioblastoma are macrophages, microglia, dendritic cells, T cells, NK cells, B cells, and, in some cases, neutrophils. Most of these populations include several subtypes or states, leading to a highly diverse and heterogeneous environment. Discussing the complex interactions between tumor cells and the immune system requires a detailed examination of the distinct phenotypes of these cell conglomerates.

### 3.1 Macrophages/microglia

**Figure 3:**
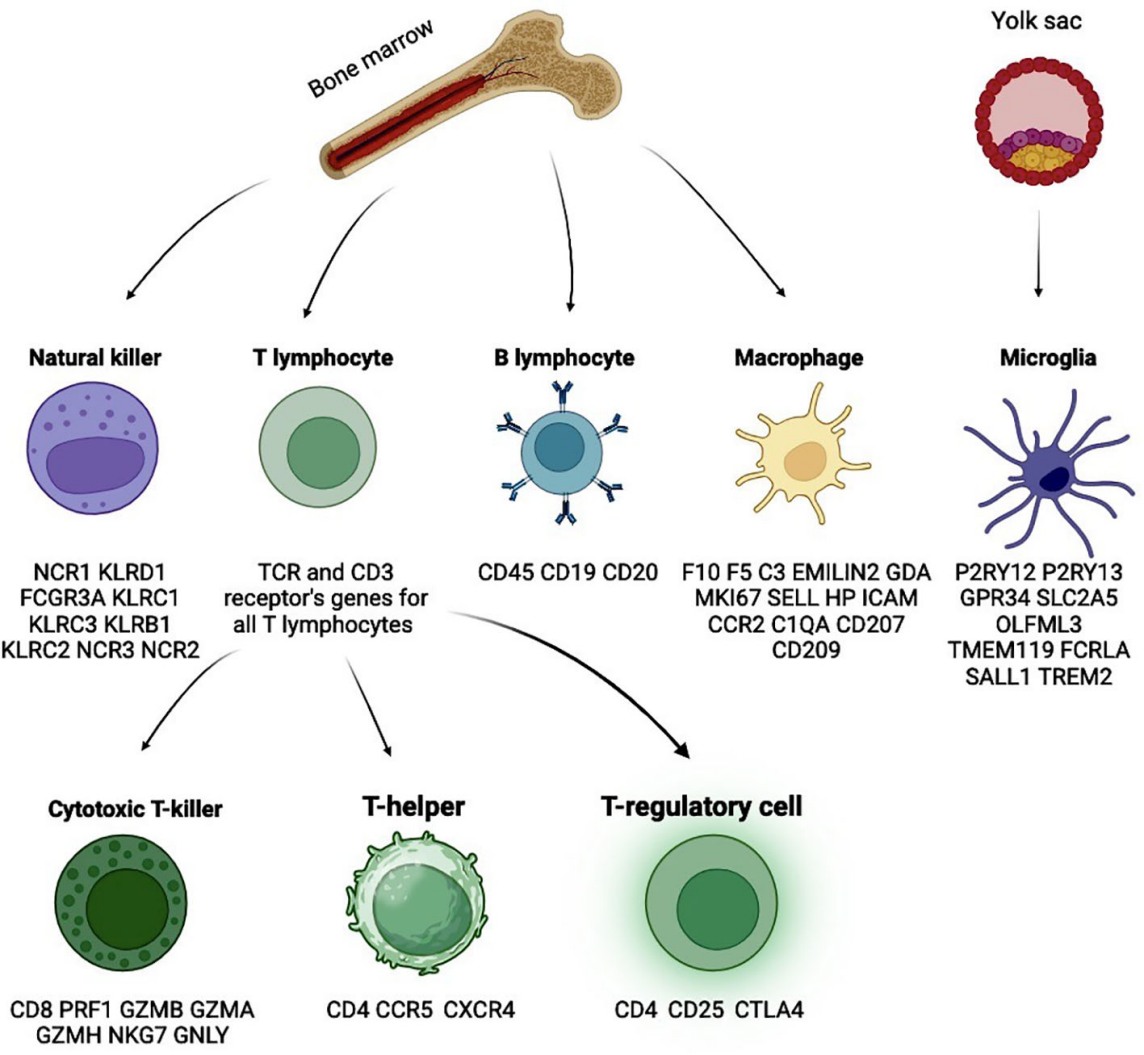
Glioblastoma microenvironment is also highly variable, reflecting the ability of tumors to reprogram stromal cells in a variety of ways. Main cell types with characteristic markers.

Among macrophages, it is important to distinguish between resident elements – microglia, and macrophages derived from blood monocytes. Microglia is a key stationary component of the central nervous system (CNS) and constitute a significant portion of its cells, originating from the embryonic yolk sac (**Figure 3**). Pathological conditions involving microglia are associated with severe neurodegenerative diseases (33). In contrast to normal physiology, some microglial cells acquire a pro-inflammatory phenotype in tumor contexts, producing signaling molecules such as IL-1α, IL-1β, TNF, and CXCL10 (34). This transformation can be initiated by tumor cells through TGFβ signaling. Specifically, pro-inflammatory microglia secrete IL-1β via the assembly of the NLRP1 inflammasome, activated by apolipoprotein E, thereby contributing to disease progression. Disruption of TGFβ signaling reduces the population of pro-inflammatory microglia and slows tumor growth (35). In murine models, cancer-mediated activation of mTORC1 in microglia has been shown to induce secretion of the anti-inflammatory cytokines IL10 and IL6. This anti-inflammatory signaling inhibits T cell infiltration into the tumor, thereby promoting tumor growth (36). Another study highlighted the role of selectins, which are involved in the anti-cancer immune response modulation. P-selectin mediates enhanced proliferation and invasion of glioblastoma by altering the activation state of microglia and macrophages. Pharmacological inhibition of P-selectin results in reduced tumor growth and increased survival in rodent models of glioblastoma (37). Murine glioblastoma models have shown tumor-induced reprogramming of microglia, with a reduction in the expression of genes promoting tumor cell destruction and an increase in the expression of genes supporting tumor growth (38). Microglial cells are predominant in newly diagnosed glioblastoma, while macrophages prevail in recurrent glioblastoma (39). Compared to the GBM IDH-mutant phenotype, microglia in GBM IDH-wildtype are characterized by the expression of *CD14* and *CD64* (40). The advent of single-cell RNA sequencing has expanded the understanding of microglial heterogeneity due to its ability to detect rare cell populations. One such population of microglia expressed genes encoding MHC I and MHC II, while another population expressed genes related to cell proliferation (*CDK1*, *STMN1*, *TUBA1b*, *TUBB5*, and *TOP2A*). Microglia with high MHC II expression have more genes coding for chemokines (*CCL3*, *CCL4*, *CCL12*), suggesting that this subset of microglia may contribute to the recruitment of other immune cells (41). A new population of immunosuppressive microglia, CD163^+^HMOX1^+^, was also identified, exhibiting anti-T cell activity through the secretion of IL-10, and this population is found to be limited in mesenchymal glioblastomas (42).

Macrophages are tissue monocytes originating from myeloid progenitors in the red bone marrow. Traditionally, macrophages are categorized into M1, the pro-inflammatory subset, and M2, the anti-inflammatory subset. However, it is challenging to clearly distinguish between these two populations, as tumor-associated macrophages (TAMs) often express markers from both groups. It is believed that in the early stages of disease, M1 macrophages predominate in tumors and limit their growth. However, tumors can recruit macrophages through a process known as M2 polarization. The M1/M2 ratio shifts towards M2 macrophages as the disease progresses, negatively impacting survival (43, 44). M1 macrophages are typically activated by interferon-gamma (IFN-γ), lipopolysaccharides from Gram-negative bacteria, tumor necrosis factor-alpha (TNF-α), and Toll-like receptors 2 and 4 (TLR2/4). M1 macrophages can induce differentiation of T cells into type 1 helper phenotype (Th1) by secreting IL-12 and activate NK cells by producing pro-inflammatory cytokines such as TNF-α, IL-1β, IL-6, IL-8, and IL-23, thereby stimulating cytotoxic responses (45–48). M2 macrophages are activated via the peroxisome proliferator-activated receptor gamma (PPARγ) and STAT6 (49, 50). The activity of the M2 macrophage subset is associated with the stimulation of Th2 T cells and regulatory T cells. M2 macrophages produce IL-1RA, IL-10, vascular endothelial growth factor (VEGF), and transforming growth factor-beta (TGF-β), which promote tumor growth (46, 48, 49). The M1/M2 polarization process correlates with the activation of several major signaling pathways, including JNK, PI3K/Akt, Notch, and JAK/STAT (50, 51). In glioma macrophages, *CCL3* is a marker of the anti-tumor fraction, while *CD68* and *CD163* are markers of the pro-tumor fraction (52). The number of CD204^+^ macrophages increases with the malignancy grade of glioma and may contribute to the pro-tumor transformation of the glioma microenvironment. CD204^+^ macrophages co-express *MMP14* and *HIF-1α*, factors potentially linked to the aggressiveness of glioma. In Grade III-IV gliomas, *CD204* expression is associated with poor prognosis (53). Glioblastoma cells can produce Wnt-induced signaling protein 1 (WISP-1), thus promoting a pro-tumor microenvironment by enhancing the survival of glioblastoma cells on one side and tumor-associated macrophages on the other (54). By synthesizing colony-stimulating factor (CSF2), glioma cells promote M2 polarization of macrophages and, consequently, tumor growth (55). Another protein supporting M2 macrophages is osteopontin (*SPP1*). The level *SPP1* expression correlates with glioma malignancy grade and the level of macrophage infiltration. In contrast, low osteopontin levels are associated with low levels of M2 macrophages and high levels of CD8^+^ cytotoxic cells, which have anti-tumor activity (56). CD11b^+^/CD163^+^ macrophages can stimulate the growth and survival of glioblastoma cells through PTN-PTPRZ1 signaling (57). CECR1 is a potential protein regulating M2 polarization of macrophages and is widely synthesized by these macrophages. It is suggested that this protein may stimulate migration and proliferation processes in glioblastoma cells through MAPK signaling (58).

As mentioned earlier, distinguishing between M1 and M2 macrophage subpopulations is a challenging. M1 macrophages are typically characterized by the expression of *CD64* (*FCGR1A*), *SOCS1*, *IL1R1*, *TLR2*, *TLR4*, *CSF2* (*GM-CSF*), *CD169* (*SIGLEC1*), *CD80*, *CD86*, *HLA-DR*, and *CD197* (*CCR7*), while M2 macrophages are marked by the hyperexpression of *MRC1* (*CD206*), *TGM2*, *CCL22*, *TLR1*, *TLR8*, *FCGR3A* (*CD16*), *CD200R1*, *MSR1* (*CD204*), *CD163*, *CD209*, and *CCL2*. There are also significant differences in the secreted biomolecules: M1 macrophages typically produce ROS, iNOS, TNF-α, IL-1β, high levels of IL-6, IL-12, IL-23, and low levels of IL-10; whereas M2 macrophages are characterized by the secretion of IL-8, high levels of IL-10, Arg-1, CCL2, CCL5, CCL17, CCL22, PDGF, VEGF, and MMPs (48, 50, 59). Distinguishing microglia from macrophages is also problematic due to the expression of classical markers for these cell populations, which may be significantly altered during oncopathophysiological reprogramming. For example, classical markers used to identify macrophages, such as CD45 and CCR2, can start being expressed by microglial cells in brain gliomas. In order to discriminate them, microglia can be identified by the expression of typical genes such as *P2RY13, P2RY12, GPR34, SLC2A5, OLFML3, TMEM119, FCRLA, SALL1*, and *TREM2*. In contrast, macrophages can be identified using markers such as *F10, EMILIN2, F5, C3, GDA, MKI67, SELL, HP, ICAM1, CCR2, C1QA, CD207*, and *CD209* (**Table 1**) (39, 41, 60–65).

### 3.2 B cells

B lymphocytes are a distinct class of immune cells, the main characteristic of which is the presence of specific receptors for antigens on their surface. These receptors are based on immunoglobulins, complex molecules that are specific to a single antigen. After interacting with the antigen, the B cell exits the bloodstream and differentiates into a plasma cell that produces immunoglobulins (antibodies) against the recognized antigen. Thus, B lymphocytes are the primary cellular substrate for humoral immunity. The role of these cells in oncology is controversial: according to some data, they have a pro-tumor effect, while other data suggest they have an anti-tumor effect (66). According to other data, glioma cells can induce the differentiation of naive B lymphocytes into TGF-β ^+^ B regulatory cells through the synthesis of PlGF. In turn, B regulatory cells, similarly to the mechanism described above, can inhibit anti-cancer T cell immunity (67). Lee-Chang C et al. demonstrated the dual nature of B cells as local elimination of tumor-associated B lymphocytes in implanted human glioblastomas in mice via anti-CD20 immunotherapy (intracranial injection) nearly doubled the survival of the mice. In contrast, its systemic administration via intraperitoneal injection showed no benefit. This observation helps explain the dual role of these cells depending on their location. After implantation of glioblastoma tissue in mice, there was an increase in B lymphocyte infiltration (mainly in perivascular areas) of the tumor stroma, which correlated with overall B lymphopenia. Within the glioblastoma, B cells appear to exert an immunosuppressive effect, while systemically, they possess anti-tumor properties. This pro-tumoral effect is mediated through the synthesis of inhibitory molecules such as PD-L1 and CD155 and immune-regulatory cytokines like TGFβ and IL-10. The result, as seen in other cases, is the differentiation of naive B cells into mature B regulatory cells and subsequent suppression of CD8^+^ cytotoxic T cells (by inhibiting the expression of *GZMB* and *IFNγ* by these cells) (68, 69). Another study demonstrated the pro-inflammatory role of so-called 4-1BBL^+^ B lymphocytes, which exhibit high intracellular levels of TNF and IFNγ, as well as increased expression of genes *CD86* and *CD69*, indicating their activated state. The level of these B lymphocytes positively correlates with the level of CD69^+^CD8^+^ T lymphocytes. The ability of B cells to stimulate CD8^+^ T cells largely depends on 4-1BBL expression. The increase in 4-1BBL levels in B cells occurs after stimulation of the B-cell receptor (BCR) and co-stimulation of CD40 (70, 71). Upon local (intracranial) injection of 4-1BBL^+^ B lymphocytes in mice with implanted glioblastoma, an increase in tumor infiltration by CD8^+^ T lymphocytes producing GzmB and IFNγ was observed. These mice lived longer than the control group (71). Thus, the role of B lymphocytes in glioblastoma pathogenesis may be of great importance. Tumor cells appear to recruit B cells, promoting their differentiation into immunosuppressive B regulatory lymphocytes, thereby supporting tumor survival and immune evasion through suppression of the T cell response. As demonstrated by the experiments of Lee-Chang C et al., activated B lymphocytes could be a potential target for glioblastoma immunotherapy. B lymphocytes are identified as CD45^+^CD11b^−^CD19^+^CD20^+^ cells.

### 3.3 T cells

T cells are a subpopulation of lymphocytes derived from hematopoietic stem cells in the bone marrow. The distinguishing feature of T lymphocytes is their unique mechanism of antigen recognition. They recognize peptide fragments of foreign proteins that are embedded in autologous major histocompatibility complex (MHC) molecules. This molecular complex is presented to them by antigen-presenting cells (such as macrophages and dendritic cells) through interaction with the T-cell receptor (TCR). There are three main groups of T lymphocytes: T-helper cells, CD8^+^ T-killer (cytotoxic T) cells, and T-regulatory cells. All T lymphocytes share common markers, including CD3 and TCR receptors (**Table 1**) (72). While the heterogeneity of T cells extends beyond these subclusters, they represent the most significant subpopulations of interest.

#### 3.3.1 T helper cells

CD4^+^ T helper cells recognize antigens presented in association with MHC II complexes. At least three types of CD4^+^ cells have been identified: T helper 1 (Th1), T helper 2 (Th2), and T helper 17 (Th17) cells. CD4+ T cells broadly express CCR5 and CXCR4 chemokine receptors, which are important due to their role as targets for HIV. Th1 cells are involved in enhancing the expression of MHC II on antigen-presenting cells, activating macrophages and B cells through the synthesis of interferon-gamma (IFN-γ). The activated macrophages, in turn, secrete IL-12, which drives the differentiation of naive T cells into Th1 cells, thereby amplifying the immune response. Th2 cells are primarily active in response to parasitic infections and promote the migration of eosinophils and mast cells by secreting IL-4, IL-5, and IL-13 (73, 74). Th17 cells play a crucial role in protecting mucosal surfaces against bacterial and fungal pathogens, and they activate neutrophils and monocytes through the secretion of IL-17A, IL-17F, and IL-22 (75).

#### 3.3.2 T regulatory cells

This subset of T cells, although part of the CD4^+^ lineage, is often categorized separately due to its specific immunosuppressive functions. The primary role of CD4^+^ regulatory T cells (Tregs) is to suppress excessive immune responses. Tregs can inhibit the differentiation of Th1 and Th17 cells through the secretion of IL-35 and suppress the activity of CD8^+^ cytotoxic T lymphocytes via IL-10 (76). The contribution of this axis in glioblastoma is not well studied, reflecting the uncertainties about the true nature of IL-35 (77). However, resent studies suggest that IL-35 plays a much more extensive role in immune suppression than previously appreciated (77, 78). Tregs also express the CTLA-4 receptor on their surface, which, when interacting with membrane proteins on antigen-presenting cells, mediates immunosuppressive effects. Key markers of Tregs include *CD25*, a receptor for IL-2 (a cytokine that activates Tregs), and the transcription factor FOXP3, which is crucial for their development and function (79).

#### 3.3.3 T killer (cytotoxic T) cells

CD8^+^ cytotoxic T lymphocytes (CTLs) recognize antigens presented in association with MHC I complexes on the surface of antigen-presenting cells. These CTLs are involved in the lysis or apoptosis-mediated elimination of cells infected with intracellular pathogens or tumor cells. Since cytotoxic T cells can only interact with antigens in the context of MHC I, tumor cells that lack this complex become invisible to CD8^+^ cells. The primary functions of CD8^+^ T cells are mediated through one of two mechanisms: either by inducing apoptosis or by lysing the target cell through the formation of pores in the membrane (via perforins) and by injecting toxic compounds (such as granzymes) into the cell. These cells mature under the influence of IL-2 (72, 79). Key markers for CD8^+^ T cells include *PRF1* (perforin)*, GZMB* (granzyme B)*, GZMA* (granzyme A)*, GZMH* (granzyme H)*, NKG7*, and *GNLY* (granulysin).

Glioblastoma has traditionally been considered a “cold” tumor due to its immunosuppressive microenvironment. Since T cell responses play a significant role in the antitumor immune defense, it is believed that increased T cell infiltration into glioblastomas could have a positive impact on prognosis and immunotherapy outcomes for this disease (80). For instance, tumors classified as immunologically “hot” are characterized by extensive infiltration of CD8^+^ lymphocytes and cells secreting pro-inflammatory cytokines, which leads to significantly better outcomes with immunotherapy (81). T cells infiltrating gliomas often express inhibitory genes such as *PDCD1*, *CTLA4*, and *LAG3* (82). Antibody-mediated blockade of PD-1/CTLA-4 enhances the antitumor activity of CD8^+^ lymphocytes (83, 84). This type of immunotherapy has shown success in treating several cancer types (85, 86). However, the use of anti-PD-1 antibodies in recurrent glioblastoma has not yielded significant results (87, 88). Another target for immunotherapy is the *KLRB1* gene, which produces the CD161 receptor on NK cells and CD8^+^ lymphocytes. *CLEC2D*, a gene that encodes a ligand for the CD161 receptor, is expressed by tumor cells and myeloid cells (microglia/macrophages). Activation of the CD161 receptor through ligand-receptor interactions may lead to reduced antitumor function of CD8^+^ cells (89).

### 3.4 NK (natural killer) cells

Natural killer (NK) cells are a crucial component of the innate immune system and act as the first line of defense against oncological diseases. A key distinguishing feature of NK cells is their ability to induce target cell lysis without prior antigen contact or activation (90). NK cells constitute approximately 10-15% of the lymphocytes in peripheral blood. Like CD8^+^ T cells, NK cells contain intracellular granules with perforin – a protein that forms pores in target cell membranes – and granzymes, which induce apoptosis. As mentioned above, tumor cells may downregulate the expression of MHC class I molecules to evade cytotoxic effects from CD8^+^ T cells. However, this mechanism can lead to NK cell activation, as NK cells express inhibitory receptors that interact with MHC I proteins to suppress their activation (91, 92). To facilitate such interactions, NK cells possess immunoglobulin-like receptors known as Killer Immunoglobulin-like Receptors (KIRs) on their surface. These receptors can engage with various forms of MHC class I molecules, such as HLA-A, HLA-B, and HLA-C. Another receptor that inhibits the cytotoxic activity of NK cells is the CD94/NKG2A complex, which specifically interacts with the HLA-E variant of MHC class I (93). Thus, the recognition of MHC class I proteins inhibits the activity of natural killer (NK) cells.

Additionally, virus-infected or tumor cells may express non-classical MHC class I molecules, such as MICA and MICB. Interaction of these molecules with the activating receptor NKG2D on NK cells can also trigger NK cell activation and subsequent cytolysis (94). Traditionally, natural killer (NK) cells are divided into two major subsets: CD56-dim/CD16^+^ and CD56-bright/CD16^−^. The first subset (referred to as NK1) has low expression of *CD56* but high expression of *CD16*. NK1 cells constitute the majority of the NK cell population and exhibit pronounced cytotoxic activity. Due to their cytotoxic nature, NK1 cells express genes essential for this function, including *GZMB, PRF, EMP3, ITGB2*, and *EB2*. In contrast, the second subset (NK2) makes up a smaller proportion of NK cells and is characterized by high cytokine production and regulatory functions. NK2 cells are distinguished by their expression of *XCL1* (95, 96). A reliable marker for identifying NK cells is *NCR1* (*NKp46*) (66). Other potential markers include *KLRD1* (*CD94*), *FCGR3A*, *KLRC1, KLRC3, KLRB1, KLRC2, NCR3 (NKp30)*, and *NCR2 (NKp44)* (**Table 1**). Chemokine receptors (CCR2, CCR5, CXCR3, and CX3CR1) on NK cells contribute to the anti-inflammatory response and the migration of these cells to sites of inflammation in response to CCL2, CCL3, CCL5, CCL8, CCL9, CCL11, CCL13, CXCL9, CXCL10, CXCL11, and CX3CL1. High infiltration of NK cells in renal cancer correlates with a positive prognosis (97). Glioblastoma cells can downregulate the expression of the activating NK cell receptor NKG2D through a TGFβ-dependent pathway, confirming the significant role of TGFβ as a key molecular mechanism for inhibiting the immune response by glial cells (98, 99). On the other hand, glioma cells exhibit increased expression of MHC I molecules, which also inhibits NK cell activity via the aforementioned mechanism (100).

### 3.5 Mesenchymal stem/stromal cells and cancer-associated fibroblasts

MSCs are a type of non-malignant stromal stem cells found in many tissues. One of their important functions is to migrate to sites of injury and support regeneration. In addition to tissue-specific MSCs, there are also bone marrow-derived MSCs. MSCs can differentiate into almost any type of stromal cell, including osteocytes, chondrocytes, adipocytes, fibroblasts, etc. MSCs are typically positive for *CD90*, *CD105*, *CD73*, and negative for *CD34*, *CD45*, *CD14*, *HLA-DR*, *CD19*, *CD79α*, and *CD11b*. Tumor cells can initiate genetic remodeling of MSCs, converting them into cancer-associated MSCs (CA-MSCs). Glioblastoma cells can recruit MSCs by secreting IL-8, TGF-ss1, NT-3 and VEGF. Evidence suggests that CA-MSCs play a key role in the formation of the TME in various cancers. CA-MSCs and their derivatives are capable of initiating angiogenesis, remodeling the TME, enhancing glioblastoma cell invasiveness, supporting tumor growth, protecting tumor cells from apoptosis, attracting immune cells and reprogramming them toward immunosuppression, maintaining and promoting GSCs differentiation, and initiating resistance to chemotherapy (101–108).

**Figure 4:**
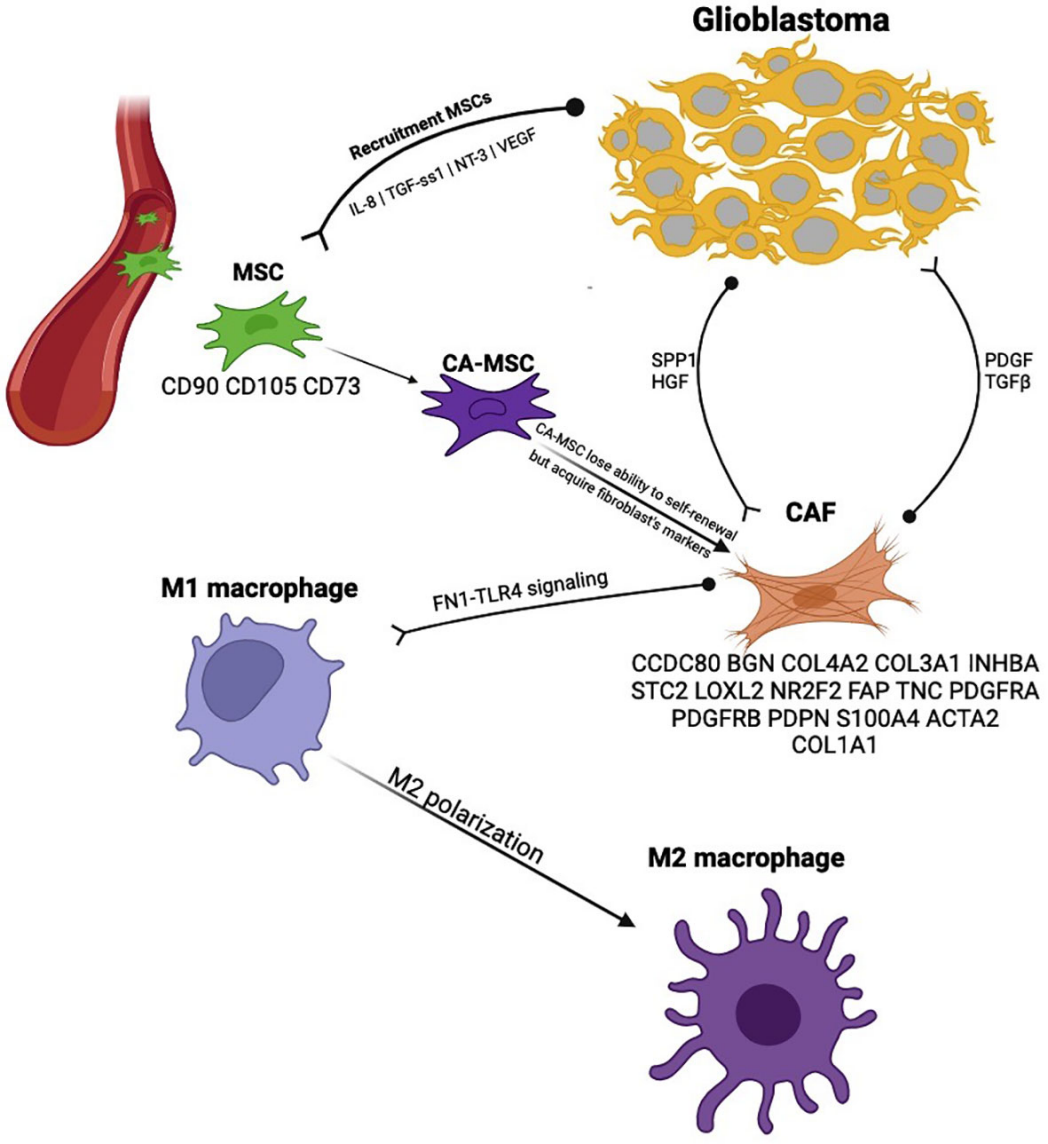
Cancer-associated fibroblasts in the glioblastoma microenvironment provide an example of specific mechanisms that are pro-tumorigenic.

Fibroblasts are an essential component of connective tissue cells. They synthesize the extracellular matrix (glycoproteins, proteoglycans, and hyaluronic acid), elastin, and collagen. Cancer-associated fibroblasts (CAFs) have been identified in the stroma of many tumors and are critical to disease progression (109) (**Figure 4**). For a long time, CAFs were thought to be absent in glioblastomas due to the lack of fibroblasts in brain tissue. However, cell populations expressing fibroblast markers have been described in gliomas (110–112). CA-MSCs are considered precursor cells for CAFs. Unlike CA-MSCs, CAFs lose the ability for self-renewal but acquire genetic markers of fibroblasts (102, 112). The expression level of CAF markers in gliomas positively correlates with tumor grade and serves as a poor prognostic indicator. CAFs appear to promote the transformation of the glioblastoma phenotype into a mesenchymal subtype (113).

The role of CAFs in M2 polarization of macrophages in gliomas has been demonstrated through IGFBP2 expression (114). M2 macrophage polarization occurs through fibronectin (FN1) and its interaction with the TLR4 receptor on macrophage surfaces. CAFs can enrich the GSCs population through the synthesis of HGF and osteopontin. In turn, GSCs affect CAFs via PDGF and TGF-β. Furthermore, adding CAFs to a GSC population *in vitro* promoted tumor growth. The expression of LRP10 in GBM correlates with the extent of tumor stroma infiltration by CAFs, and the knockdown of LRP10 significantly restricted the invasive behavior of GBM (115). Saket Jain et al. identified 18 markers of CAFs: *ACTA2, FAP, PDGFRA, PDGFRB, PDPN, S100A4, TNC, VIM, COL1A1, CCDC80, BGN, COL4A2, COL5A1, NR2F2, COL3A1, INHBA, STC2*, and *LOXL2* (116). However, according to Wu Lili et al. the most reliable CAF markers in GBM are *ACTA2*, *COL1A1*, and *TNC* (**Table 1**) (115).

## 4 Cell communications in glioma microenvironment

The transition from unicellular to multicellular forms of life marked a significant biological revolution, necessitating cellular upgrades on multiple levels, including ensuring the coordinated and harmonious functioning of all cells within a single organism. One of the cornerstones of this process is a ligand-receptor interaction (LRI). The secretion of ligands by effector cells and their interaction with receptors on the surface of target cells provide basis of intercellular communication. Through LRI, glioma cells can influence their microenvironment to recruit immune cells to support their growth and proliferation.

**Figure 5:**
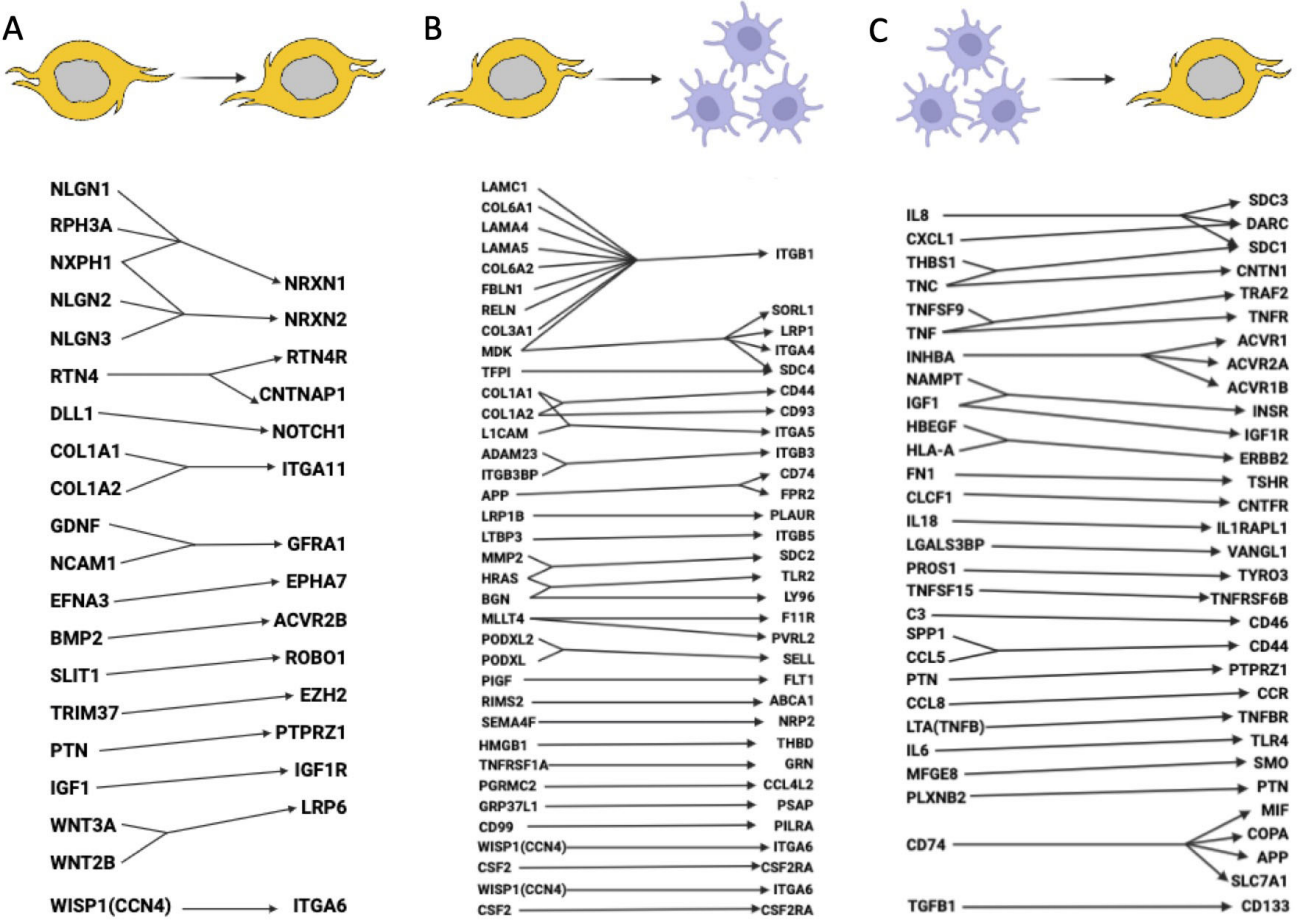
Ligand-receptor interactions between glioma cells and macrophages. **(A)** Signaling pathways within glioma cells (glioma cells express both the ligand and receptor). **(B)** Signaling pathways between glioma cells and macrophages (glioma cells express the ligand, macrophages express the receptor). **(C)** Signaling pathways between macrophages and glioma cells (macrophages express the ligand, glioma cells express the receptor). It is likely that many other important ligand-receptor pairs await validation.

Single-cell sequencing assisted in revealing numerous cell-to-cell communications in the glioma microenvironment (30, 34, 39, 41, 60, 61, 65, 80, 116–121). We compiled the most important interactions in **Figure 5**, but we can only capture those receptor-ligand pairs that have been validated at this time.

scRNA-seq data analysis using manifold learning revealed various ligand-receptor interactions, including autocrine signaling, interactions between GSCs and macrophages, and reverse macrophage-tumor interactions (117). Overall, the study revealed more than 60 LRI, which are presented in **Figure 5**. The signaling pathways by which GSCs recruit TAMs to the tumor microenvironment and promote their polarization into tumor-promoting macrophages is reviewed in (118) and are also added to **Figure 5**. An integrated analysis of 201,986 human glioma, immune, and other stromal cells confirmed heterogeneity of tumor microenvironment for gliomas and revealed S100A4 as a regulator of immune suppressive T and myeloid cells in glioblastoma (120). Glioma-myeloid and myeloid-glioma signaling identified EGFR, SPP1, MIF, and other important LRI (**Figure 5**). 86 glioblastoma samples with scRNA-seq, single-cell open-chromatin, DNA and spatial transcriptomic and proteomic assays were aggregated to study glioblastoma evolution under therapy. The authors found that recurrent GBMs are characterized by a shift to a mesenchymal phenotype (122). Important LRI between GBM cell types are included in **Figure 5**.

High macrophage signaling, as compared to other cells, was found in (121). Among the high LRI were SPP1-CD44 within macrophages; VEGFB-VEGFR1 and ANXA1-FPR1 for macrophages interacted with other non-malignant cells. Notch signaling pathways may have different roles in various cancer types, acting as both an oncogenic factor and a tumor suppressor (123). Elevated expression of *NOTCH1* is associated with advanced glioma grade and poor prognosis (124). Single-cell spatial transcriptomics analysis revealed increased expression of notch signaling in malignant cells residing in infiltrated brain tissue (119) (**Figure 5**). Overexpression of TRIM37 that maintains the cell growth and stemness in GSCs through the interaction with EZH2 was found in gliomas (125) (**Figure 5**). EZH2 activates sonic hedgehog pathway by epigenetically PTCH1 downregulation.

Of specific interest is a collagen landscape in solid tumors as they are specific to CAFs (126). It has been found that GSCs highly express collagen I/III genes such as *COL1A1, COL1A2, COL3A1, COL6A1*, and *COL6A2*. These genes may help GSCs migrate and remain mobile within the tumor. Additionally, a connection has been identified between GSCs and macrophages through the PLGF ligand, secreted by tumor cells, and its sole receptor VEGFR1, which is synthesized by macrophages (117). MAPK signaling pathway genes, known as associated with tumor development in many cancers, are also overexpressed in glioma (127). Current treatment options related to all known upregulated signaling pathways in gliomas are reviewed in (4).

## 5 Future directions

Glioblastoma is one of the most aggressive and difficult-to-treat cancers, reflecting the genetic and phenotypic plasticity of the lineage from which they arise. Despite the use of combined treatment approaches, including radical surgery, chemotherapy, and radiation therapy, the average patient survival time does not exceed 12-18 months. In some cases, the location of the tumor in functionally significant areas of the central nervous system precludes the use of surgical treatment, further shortening the already limited lifespan of patients with this diagnosis. This underscores the urgent need for new treatment strategies. One promising avenue in this regard is immunotherapy. Other potential approaches include strategies to drive the differentiation of stem cells as demonstrated by the use of retinoic acid to treat acute promyelocytic leukemias.

The immunotherapy approach is challenging. Glioblastoma exhibits remarkably multifaceted mechanisms of immune evasion and is characterized as an extremely heterogeneous tumor. This heterogeneity is evident both within the GBM itself, which can display several different phenotypes within a single tumor, and in its microenvironment. Such heterogeneity evidences the underlying phenotypic plasticity of the tumor that complicates treatment and necessitates the development of more precise therapeutic approaches that target the cancer stem cell.

Another crucial characteristic of glioblastoma is its “cold” microenvironment, which is marked by significant immunosuppression. The GBM microenvironment contains few cytotoxic cells capable of killing tumor cells and is rich in immunosuppressive components, including Tregs, Bregs, macrophages, and CAFs. This poses an additional challenge to the effective use of traditional treatments, particularly immunotherapy, which relies on the active participation of the immune system to combat the tumor. In contrast, “hot” tumors with high immune reactivity generally have a more favorable prognosis and respond better to immunotherapy. The immunosuppressive nature of glioblastoma arises from a cascade of signaling interactions between tumor cells and the surrounding microenvironment, ultimately driving immune cells toward an anti-inflammatory phenotype. Under normal conditions, immunological homeostasis is maintained through highly regulated molecular interactions between various immune cells (128). Disruption of this balance can lead to significant pathophysiological reorganization of the tumor microenvironment. Consequently, one strategy could involve modifying the GBM microenvironment to make it “hot” and enhance the effectiveness of immunotherapy. In the experiments by Lee-Chang et al. (71) on mice, significant advancements were demonstrated in enhancing the immunoreactivity of the glioblastoma microenvironment through intracranial injection of activated 4-1BBL^+^ B-lymphocytes or anti-CD20 antibodies targeting immunosuppressive regulatory B lymphocytes. These approaches promoted the infiltration of CD8^+^ cytotoxic T cells into the tumor, leading to improved survival in mice. Such studies may yield greater benefits, as combination therapy is potentially more effective than monotherapy. A significant limitation of immunotherapy is the unpredictable response of the immune system to combinatorial immunotherapy, further compounded by tumor plasticity, immune evasion mechanisms, and resistance to checkpoint blockade therapy. Mouse models are particularly useful in this context as they allow researchers to bypass these concerns. However, these experiments have species-specific limitations and cannot be directly extrapolated to humans, they remain valuable for testing various hypotheses.

Alternative treatment methods include so-called reprogramming technologies, which can transform tumor cells into terminally differentiated cells. It is believed that GSCs originate from NSCs, which can differentiate into glial or neuronal progenitors. Several potential signaling pathways capable of inducing such an effect have been identified. For example, ZNF117 controls the differentiation of GSCs towards the oligodendroglial lineage, while NeuroD4 and CP-673451 can trigger differentiation of GSCs into neuron-like cells, as well as reduce proliferation and invasion of tumor cells (129–131). These approaches are quite promising but also face the challenge of targeted impact. However, a highly effective treatment that results in a 10-year survival rate exceeding 95% for the otherwise rapidly fatal acute promyelocytic leukemia involves the systemic administration of retinoic acid and arsenic trioxide to induce differentiation of leukemia stem cells (132). In the future, such therapies should be investigated and integrated with other approaches to achieve maximum effectiveness.

A major challenge in glioblastoma treatment is the identification of therapeutic targets. This review outlines various mechanisms of immune suppression within the TME and tumor cell survival. The accumulated data suggests the need for patient-individualized poly-immunotherapy, as the heterogeneity of glioblastoma significantly limits the effectiveness of universal treatment strategies currently employed. Recent studies utilizing single-cell RNA sequencing have shown promising results, revealing previously unknown characteristics of such tumors and potentially unmasking their vulnerabilities. Advancing such approaches could be a pivotal step toward improving outcomes for glioblastoma patients and unlocking new therapeutic opportunities, including those that overcome the proliferative potential by inducing differentiation of the cancer stem cells or by preventing reprogramming of stromal cells. Investigating the TME of individual patients through single-cell transcriptomic analysis, followed by the application of a tailored cocktail that exploits a particular tumor’s vulnerabilities, may hold the key to extending the survival of patients with this aggressive cancer.

## 6 Conclusion

In this review, we explored key aspects of glioblastoma biology, focusing on its origin, classification, and microenvironmental interactions. We analyzed the potential cellular progenitors of glioblastoma, highlighting critical insights from single-cell classifications. A comprehensive overview of glioblastoma subtypes was provided, consolidating known molecular markers. Furthermore, we characterized the diverse cellular components of the tumor microenvironment, detailing their functional roles and genetic signatures. We also examined ligand-receptor interactions within glioblastoma, shedding light on the complex molecular crosstalk that shapes tumor progression. Finally, we proposed a research framework to guide future studies, aiming to refine our understanding of glioblastoma biology and advance therapeutic strategies.

The glioblastoma microenvironment remains insufficiently studied and requires further investigation. Understanding the interplay between glioblastoma cells and their surrounding microenvironment will be key to developing innovative therapeutic strategies that can improve patient survival. Inducing the differentiation of glioma stem cells to counter their plasticity and prevent their proliferation seems very promising. Research directions focused on the discovery of new immunological checkpoints are also perspective in terms of shifting the tumor microenvironment from an immunosuppressive to an immune-reactive state. The rapid course of glioblastomas reasons quick implementation of this class of therapeutic in preclinical trials. Here we also pay special attention to GSCs populations and their influence on the microenvironment. More specifically, their role in reprogramming regulatory T- and B-lymphocytes, macrophages, and cancer-associated fibroblasts is currently not well understood. Concluding, synergy of immune checkpoint inhibitors, adoptive cell therapies, and novel differentiation-inducing agents could further contribute to a more effective and durable therapeutic response.

## References

[1] Thakkar JP Dolecek TA Horbinski C Ostrom QT Lightner DD Barnholtz-Sloan JS. Epidemiologic and molecular prognostic review of glioblastoma. Cancer Epidemiol Biomarkers Prev. (2014) 23:1985–96. doi: 10.1158/1055-9965.EPI-14-0275 PMC4185005 25053711

[2] Grech N Dalli T Mizzi S Meilak L Calleja N Zrinzo A. Rising incidence of glioblastoma multiforme in a well-defined population. Cureus. (2020) 12:e8195. doi: 10.7759/cureus.8195 32572354 PMC7302718

[3] Osborn AG Louis DN Poussaint TY Linscott LL Salzman KL. The 2021 world health organization classification of tumors of the central nervous system: what neuroradiologists need to know. AJNR Am J Neuroradiol. (2022) 43:928–37. doi: 10.3174/ajnr.A7462 PMC9262075 35710121

[4] Dewdney B Jenkins MR Best SA Freytag S Prasad K Holst J. From signalling pathways to targeted therapies: unravelling glioblastoma’s secrets and harnessing two decades of progress. Signal Transduct Target Ther. (2023) 8:400. doi: 10.1038/s41392-023-01637-8 37857607 PMC10587102

[5] Lee JH Lee JE Kahng JY Kim SH Park JS Yoon SJ. Human glioblastoma arises from subventricular zone cells with low-level driver mutations. Nature. (2018) 560:243–7. doi: 10.1038/s41586-018-0389-3 30069053

[6] Yao M Li S Wu X Diao S Zhang G He H. Cellular origin of glioblastoma and its implication in precision therapy. Cell Mol Immunol. (2018) 15:737–9. doi: 10.1038/cmi.2017.159 PMC6141605 29553137

[7] Lu C Garipler G Dai C Roush T Salome-Correa J Martin A. Essential transcription factors for induced neuron differentiation. Nat Commun. (2023) 14:8362. doi: 10.1038/s41467-023-43602-7 38102126 PMC10724217

[8] Tiane A Schepers M Rombaut B Hupperts R Prickaerts J Hellings N. From opc to oligodendrocyte: an epigenetic journey. Cells. (2019) 8:1–19. doi: 10.3390/cells8101236 PMC6830107 31614602

[9] Siletti K Hodge R Mossi Albiach A Lee KW Ding SL Hu L. Transcriptomic diversity of cell types across the adult human brain. Science. (2023) 382:eadd7046. doi: 10.1126/science.add7046 37824663

[10] Verhaak RG Hoadley KA Purdom E Wang V Qi Y Wilkerson MD. Integrated genomic analysis identifies clinically relevant subtypes of glioblastoma characterized by abnormalities in pdgfra, idh1, egfr, and nf1. Cancer Cell. (2010) 17:98–110. doi: 10.1016/j.ccr.2009.12.020 20129251 PMC2818769

[11] Neftel C Laffy J Filbin MG Hara T Shore ME Rahme GJ. An integrative model of cellular states, plasticity, and genetics for glioblastoma. Cell. (2019) 178:835–49 e21. doi: 10.1016/j.cell.2019.06.024 31327527 PMC6703186

[12] Xu L Peng B Wu H Zheng Y Yu Q Fang S. Mettl7b contributes to the Malignant progression of glioblastoma by inhibiting egr1 expression. Metab Brain Dis. (2022) 37:1133–43. doi: 10.1007/s11011-022-00925-6 35254598

[13] Wang Q Hu B Hu X Kim H Squatrito M Scarpace L. Tumor evolution of glioma-intrinsic gene expression subtypes associates with immunological changes in the microenvironment. Cancer Cell. (2017) 32:42–56 e6. doi: 10.1016/j.ccell.2017.06.003 28697342 PMC5599156

[14] Varn FS Johnson KC Martinek J Huse JT Nasrallah MP Wesseling P. Glioma progression is shaped by genetic evolution and microenvironment interactions. Cell. (2022) 185:2184–99 e16. doi: 10.1016/j.cell.2022.04.038 35649412 PMC9189056

[15] Lim S Kaldis P. Loss of cdk2 and cdk4 induces a switch from proliferation to differentiation in neural stem cells. Stem Cells. (2012) 30:1509–20. doi: 10.1002/stem.1114 22532528

[16] Zhu Q Zhao X Zheng K Li H Huang H Zhang Z. Genetic evidence that nkx2.2 and pdgfra are major determinants of the timing of oligodendrocyte differentiation in the developing cns. Development. (2014) 141:548–55. doi: 10.1242/dev.095323 PMC3899813 24449836

[17] Sottoriva A Spiteri I Piccirillo SG Touloumis A Collins VP Marioni JC. Intratumor heterogeneity in human glioblastoma reflects cancer evolutionary dynamics. Proc Natl Acad Sci U.S.A. (2013) 110:4009–14. doi: 10.1073/pnas.1219747110 PMC3593922 23412337

[18] Buchwald ZS Tian S Rossi M Smith GH Switchenko J Hauenstein JE. Genomic copy number variation correlates with survival outcomes in who grade iv glioma. Sci Rep. (2020) 10:7355. doi: 10.1038/s41598-020-63789-9 32355162 PMC7192941

[19] Weller M Butowski N Tran DD Recht LD Lim M Hirte H. Rindopepimut with temozolomide for patients with newly diagnosed, egfrviii-expressing glioblastoma (Act iv): A randomised, double-blind, international phase 3 trial. Lancet Oncol. (2017) 18:1373–85. doi: 10.1016/S1470-2045(17)30517-X 28844499

[20] Lathia JD Mack SC Mulkearns-Hubert EE Valentim CL Rich JN. Cancer stem cells in glioblastoma. Genes Dev. (2015) 29:1203–17. doi: 10.1101/gad.261982.115 PMC4495393 26109046

[21] Chen J Li Y Yu TS McKay RM Burns DK Kernie SG. A restricted cell population propagates glioblastoma growth after chemotherapy. Nature. (2012) 488:522–6. doi: 10.1038/nature11287 PMC3427400 22854781

[22] Anido J Saez-Borderias A Gonzalez-Junca A Rodon L Folch G Carmona MA. Tgf-beta receptor inhibitors target the cd44(High)/id1(High) glioma-initiating cell population in human glioblastoma. Cancer Cell. (2010) 18:655–68. doi: 10.1016/j.ccr.2010.10.023 21156287

[23] Ikushima H Todo T Ino Y Takahashi M Miyazawa K Miyazono K. Autocrine tgf-beta signaling maintains tumorigenicity of glioma-initiating cells through sry-related hmg-box factors. Cell Stem Cell. (2009) 5:504–14. doi: 10.1016/j.stem.2009.08.018 19896441

[24] Wu M Guan J Li C Gunter S Nusrat L Ng S. Aberrantly activated cox-2 and wnt signaling interact to maintain cancer stem cells in glioblastoma. Oncotarget. (2017) 8:82217–30. doi: 10.18632/oncotarget.19283 PMC5669884 29137258

[25] Suva ML Rheinbay E Gillespie SM Patel AP Wakimoto H Rabkin SD. Reconstructing and reprogramming the tumor-propagating potential of glioblastoma stem-like cells. Cell. (2014) 157:580–94. doi: 10.1016/j.cell.2014.02.030 PMC4004670 24726434

[26] Brescia P Richichi C Pelicci G. Current strategies for identification of glioma stem cells: adequate or unsatisfactory? J Oncol. (2012) 2012:376894. doi: 10.1155/2012/376894 22685459 PMC3366252

[27] Suva ML Tirosh I. The glioma stem cell model in the era of single-cell genomics. Cancer Cell. (2020) 37:630–6. doi: 10.1016/j.ccell.2020.04.001 32396858

[28] Bhaduri A Di Lullo E Jung D Muller S Crouch EE Espinosa CS. Outer radial glia-like cancer stem cells contribute to heterogeneity of glioblastoma. Cell Stem Cell. (2020) 26:48–63 e6. doi: 10.1016/j.stem.2019.11.015 31901251 PMC7029801

[29] Lei Y Tang R Xu J Wang W Zhang B Liu J. Applications of single-cell sequencing in cancer research: progress and perspectives. J Hematol Oncol. (2021) 14:91. doi: 10.1186/s13045-021-01105-2 34108022 PMC8190846

[30] Xin S Liu X Li Z Sun X Wang R Zhang Z. Scrna-seq revealed an immunosuppression state and tumor microenvironment heterogeneity related to lymph node metastasis in prostate cancer. Exp Hematol Oncol. (2023) 12:49. doi: 10.1186/s40164-023-00407-0 37221625 PMC10204220

[31] de Visser KE Joyce JA. The evolving tumor microenvironment: from cancer initiation to metastatic outgrowth. Cancer Cell. (2023) 41:374–403. doi: 10.1016/j.ccell.2023.02.016 36917948

[32] Hambardzumyan D Bergers G. Glioblastoma: defining tumor niches. Trends Cancer. (2015) 1:252–65. doi: 10.1016/j.trecan.2015.10.009 PMC4831073 27088132

[33] Gao C Jiang J Tan Y Chen S. Microglia in neurodegenerative diseases: mechanism and potential therapeutic targets. Signal Transduct Target Ther. (2023) 8:359. doi: 10.1038/s41392-023-01588-0 37735487 PMC10514343

[34] Yeo AT Rawal S Delcuze B Christofides A Atayde A Strauss L. Single-cell rna sequencing reveals evolution of immune landscape during glioblastoma progression. Nat Immunol. (2022) 23:971–84. doi: 10.1038/s41590-022-01215-0 PMC9174057 35624211

[35] Liu H Sun Y Zhang Q Jin W Gordon RE Zhang Y. Pro-inflammatory and proliferative microglia drive progression of glioblastoma. Cell Rep. (2021) 36:109718. doi: 10.1016/j.celrep.2021.109718 34525361

[36] Dumas AA Pomella N Rosser G Guglielmi L Vinel C Millner TO. Microglia promote glioblastoma via mtor-mediated immunosuppression of the tumour microenvironment. EMBO J. (2020) 39:e103790. doi: 10.15252/embj.2019103790 32567735 PMC7396846

[37] Yeini E Ofek P Pozzi S Albeck N Ben-Shushan D Tiram G. P-selectin axis plays a key role in microglia immunophenotype and glioblastoma progression. Nat Commun. (2021) 12:1912. doi: 10.1038/s41467-021-22186-0 33771989 PMC7997963

[38] Maas SLN Abels ER Van De Haar LL Zhang X Morsett L Sil S. Glioblastoma hijacks microglial gene expression to support tumor growth. J Neuroinflamm. (2020) 17:120. doi: 10.1186/s12974-020-01797-2 PMC7164149 32299465

[39] Pombo Antunes AR Scheyltjens I Lodi F Messiaen J Antoranz A Duerinck J. Single-cell profiling of myeloid cells in glioblastoma across species and disease stage reveals macrophage competition and specialization. Nat Neurosci. (2021) 24:595–610. doi: 10.1038/s41593-020-00789-y 33782623

[40] Klemm F Maas RR Bowman RL Kornete M Soukup K Nassiri S. Interrogation of the microenvironmental landscape in brain tumors reveals disease-specific alterations of immune cells. Cell. (2020) 181:1643–60 e17. doi: 10.1016/j.cell.2020.05.007 32470396 PMC8558904

[41] Ochocka N Segit P Walentynowicz KA Wojnicki K Cyranowski S Swatler J. Single-cell rna sequencing reveals functional heterogeneity of glioma-associated brain macrophages. Nat Commun. (2021) 12:1151. doi: 10.1038/s41467-021-21407-w 33608526 PMC7895824

[42] Ravi VM Neidert N Will P Joseph K Maier JP Kuckelhaus J. T-cell dysfunction in the glioblastoma microenvironment is mediated by myeloid cells releasing interleukin-10. Nat Commun. (2022) 13:925. doi: 10.1038/s41467-022-28523-1 35177622 PMC8854421

[43] Gieryng A Pszczolkowska D Walentynowicz KA Rajan WD Kaminska B. Immune microenvironment of gliomas. Lab Invest. (2017) 97:498–518. doi: 10.1038/labinvest.2017.19 28287634

[44] Deng Y Chen Q Wan C Sun Y Huang F Hu Y. Microglia and macrophage metabolism: A regulator of cerebral gliomas. Cell Biosci. (2024) 14:49. doi: 10.1186/s13578-024-01231-7 38632627 PMC11022384

[45] Martinez FO Sica A Mantovani A Locati M. Macrophage activation and polarization. Front Biosci. (2008) 13:453–61. doi: 10.2741/2692 17981560

[46] Mantovani A Biswas SK Galdiero MR Sica A Locati M. Macrophage plasticity and polarization in tissue repair and remodelling. J Pathol. (2013) 229:176–85. doi: 10.1002/path.4133 23096265

[47] Hambardzumyan D Gutmann DH Kettenmann H. The role of microglia and macrophages in glioma maintenance and progression. Nat Neurosci. (2016) 19:20–7. doi: 10.1038/nn.4185 PMC4876023 26713745

[48] Cui A Huang T Li S Ma A Perez JL Sander C. Dictionary of immune responses to cytokines at single-cell resolution. Nature. (2024) 625:377–84. doi: 10.1038/s41586-023-06816-9 PMC10781646 38057668

[49] Lawrence T Natoli G. Transcriptional regulation of macrophage polarization: enabling diversity with identity. Nat Rev Immunol. (2011) 11:750–61. doi: 10.1038/nri3088 22025054

[50] Chen S Saeed A Liu Q Jiang Q Xu H Xiao GG. Macrophages in immunoregulation and therapeutics. Signal Transduct Target Ther. (2023) 8:207. doi: 10.1038/s41392-023-01452-1 37211559 PMC10200802

[51] Zhou D Huang C Lin Z Zhan S Kong L Fang C. Macrophage polarization and function with emphasis on the evolving roles of coordinated regulation of cellular signaling pathways. Cell Signal. (2014) 26:192–7. doi: 10.1016/j.cellsig.2013.11.004 24219909

[52] Vidyarthi A Agnihotri T Khan N Singh S Tewari MK Radotra BD. Predominance of M2 macrophages in gliomas leads to the suppression of local and systemic immunity. Cancer Immunol Immunother. (2019) 68:1995–2004. doi: 10.1007/s00262-019-02423-8 31690954 PMC11028103

[53] Sorensen MD Dahlrot RH Boldt HB Hansen S Kristensen BW. Tumour-associated microglia/macrophages predict poor prognosis in high-grade gliomas and correlate with an aggressive tumour subtype. Neuropathol Appl Neurobiol. (2018) 44:185–206. doi: 10.1111/nan.12428 28767130

[54] Tao W Chu C Zhou W Huang Z Zhai K Fang X. Dual role of wisp1 in maintaining glioma stem cells and tumor-supportive macrophages in glioblastoma. Nat Commun. (2020) 11:3015. doi: 10.1038/s41467-020-16827-z 32541784 PMC7295765

[55] Sielska M Przanowski P Pasierbinska M Wojnicki K Poleszak K Wojtas B. Tumour-derived csf2/granulocyte macrophage colony stimulating factor controls myeloid cell accumulation and progression of gliomas. Br J Cancer. (2020) 123:438–48. doi: 10.1038/s41416-020-0862-2 PMC7403321 32390004

[56] Wei J Marisetty A Schrand B Gabrusiewicz K Hashimoto Y Ott M. Osteopontin mediates glioblastoma-associated macrophage infiltration and is a potential therapeutic target. J Clin Invest. (2019) 129:137–49. doi: 10.1172/JCI121266 PMC6307970 30307407

[57] Shi Y Ping YF Zhou W He ZC Chen C Bian BS. Tumour-associated macrophages secrete pleiotrophin to promote ptprz1 signalling in glioblastoma stem cells for tumour growth. Nat Commun. (2017) 8:15080. doi: 10.1038/ncomms15080 28569747 PMC5461490

[58] Zhu C Mustafa D Zheng PP van der Weiden M Sacchetti A Brandt M. Activation of cecr1 in M2-like tams promotes paracrine stimulation-mediated glial tumor progression. Neuro Oncol. (2017) 19:648–59. doi: 10.1093/neuonc/now251 PMC5464467 28453746

[59] Gao L Wang FQ Li HM Yang JG Ren JG He KF. Ccl2/egf positive feedback loop between cancer cells and macrophages promotes cell migration and invasion in head and neck squamous cell carcinoma. Oncotarget. (2016) 7:87037–51. doi: 10.18632/oncotarget.13523 PMC5349969 27888616

[60] Hammond TR Dufort C Dissing-Olesen L Giera S Young A Wysoker A. Single-cell rna sequencing of microglia throughout the mouse lifespan and in the injured brain reveals complex cell-state changes. Immunity. (2019) 50:253–71 e6. doi: 10.1016/j.immuni.2018.11.004 30471926 PMC6655561

[61] Friebel E Kapolou K Unger S Nunez NG Utz S Rushing EJ. Single-cell mapping of human brain cancer reveals tumor-specific instruction of tissue-invading leukocytes. Cell. (2020) 181:1626–42 e20. doi: 10.1016/j.cell.2020.04.055 32470397

[62] Akkari L Bowman RL Tessier J Klemm F Handgraaf SM de Groot M. Dynamic changes in glioma macrophage populations after radiotherapy reveal csf-1r inhibition as a strategy to overcome resistance. Sci Transl Med. (2020) 12:1–13. doi: 10.1126/scitranslmed.aaw7843 32669424

[63] Haage V Semtner M Vidal RO Hernandez DP Pong WW Chen Z. Comprehensive gene expression meta-analysis identifies signature genes that distinguish microglia from peripheral monocytes/macrophages in health and glioma. Acta Neuropathol Commun. (2019) 7:20. doi: 10.1186/s40478-019-0665-y 30764877 PMC6376799

[64] Li Q Lan X Han X Wang J. Expression of tmem119/sall1 and ccr2/cd69 in facs-sorted microglia- and monocyte/macrophage-enriched cell populations after intracerebral hemorrhage. Front Cell Neurosci. (2018) 12:520. doi: 10.3389/fncel.2018.00520 30687011 PMC6333739

[65] Woolf Z Swanson MEV Smyth LC Mee EW Schweder P Heppner P. Single-cell image analysis reveals a protective role for microglia in glioblastoma. Neurooncol Adv. (2021) 3:vdab031. doi: 10.1093/noajnl/vdab031 34286275 PMC8284623

[66] Yuen GJ Demissie E Pillai S. B lymphocytes and cancer: A love-hate relationship. Trends Cancer. (2016) 2:747–57. doi: 10.1016/j.trecan.2016.10.010 PMC5472356 28626801

[67] Han S Feng S Ren M Ma E Wang X Xu L. Glioma cell-derived placental growth factor induces regulatory B cells. Int J Biochem Cell Biol. (2014) 57:63–8. doi: 10.1016/j.biocel.2014.10.005 25450457

[68] Lee-Chang C Rashidi A Miska J Zhang P Pituch KC Hou D. Myeloid-derived suppressive cells promote B cell-mediated immunosuppression via transfer of pd-L1 in glioblastoma. Cancer Immunol Res. (2019) 7:1928–43. doi: 10.1158/2326-6066.CIR-19-0240 PMC6891201 31530559

[69] Spallone A. Editorial: modern neurosurgical management of gliomas, including local therapies. Front Oncol. (2023) 13:1217180. doi: 10.3389/fonc.2023.1217180 37614507 PMC10443098

[70] Futagawa T Akiba H Kodama T Takeda K Hosoda Y Yagita H. Expression and function of 4-1bb and 4-1bb ligand on murine dendritic cells. Int Immunol. (2002) 14:275–86. doi: 10.1093/intimm/14.3.275 11867564

[71] Lee-Chang C Miska J Hou D Rashidi A Zhang P Burga RA. Activation of 4-1bbl+ B cells with cd40 agonism and ifngamma elicits potent immunity against glioblastoma. J Exp Med. (2021) 218:1–23. doi: 10.1084/jem.20200913 PMC7527974 32991668

[72] Sauls RS McCausland C Taylor BN. Histology, T-cell lymphocyte. USA: Statpearls Treasure Island (FL) (2024). 30571054

[73] Williams JW Ferreira CM Blaine KM Rayon C Velazquez F Tong J. Non-apoptotic fas (Cd95) signaling on T cells regulates the resolution of th2-mediated inflammation. Front Immunol. (2018) 9:2521. doi: 10.3389/fimmu.2018.02521 30443253 PMC6221963

[74] Hirata H Yukawa T Tanaka A Miyao T Fukuda T Fukushima Y. Th2 cell differentiation from naive cd4(+) T cells is enhanced by autocrine cc chemokines in atopic diseases. Clin Exp Allergy. (2019) 49:474–83. doi: 10.1111/cea.13313 30431203

[75] Batista-Duharte A Tellez-Martinez D Roberto de Andrade C Portuondo DL Jellmayer JA Polesi MC. Sporothrix brasiliensis induces a more severe disease associated with sustained th17 and regulatory T cells responses than sporothrix schenckii sensu stricto in mice. Fungal Biol. (2018) 122:1163–70. doi: 10.1016/j.funbio.2018.08.004 30449354

[76] Collison LW Workman CJ Kuo TT Boyd K Wang Y Vignali KM. The inhibitory cytokine il-35 contributes to regulatory T-cell function. Nature. (2007) 450:566–9. doi: 10.1038/nature06306 18033300

[77] Aparicio-Siegmund S Moll JM Lokau J Grusdat M Schroder J Plohn S. Recombinant P35 from bacteria can form interleukin (Il-)12, but not il-35. PloS One. (2014) 9:e107990. doi: 10.1371/journal.pone.0107990 25259790 PMC4178060

[78] Sullivan JA Tomita Y Jankowska-Gan E Lema DA Arvedson MP Nair A. Treg-cell-derived il-35-coated extracellular vesicles promote infectious tolerance. Cell Rep. (2020) 30:1039–51 e5. doi: 10.1016/j.celrep.2019.12.081 31995748 PMC7042971

[79] Jeon PH Oh KI. Il2 is required for functional maturation of regulatory T cells. Anim Cells Syst (Seoul). (2017) 21:1–9. doi: 10.1080/19768354.2016.1272489 30460045 PMC6138303

[80] Eisenbarth D Wang YA. Glioblastoma heterogeneity at single cell resolution. Oncogene. (2023) 42:2155–65. doi: 10.1038/s41388-023-02738-y PMC10913075 37277603

[81] Galon J Bruni D. Approaches to treat immune hot, altered and cold tumours with combination immunotherapies. Nat Rev Drug Discovery. (2019) 18:197–218. doi: 10.1038/s41573-018-0007-y 30610226

[82] Woroniecka K Chongsathidkiet P Rhodin K Kemeny H Dechant C Farber SH. T-cell exhaustion signatures vary with tumor type and are severe in glioblastoma. Clin Cancer Res. (2018) 24:4175–86. doi: 10.1158/1078-0432.CCR-17-1846 PMC6081269 29437767

[83] Zou W Wolchok JD Chen L. Pd-L1 (B7-H1) and pd-1 pathway blockade for cancer therapy: mechanisms, response biomarkers, and combinations. Sci Transl Med. (2016) 8:328rv4. doi: 10.1126/scitranslmed.aad7118 PMC4859220 26936508

[84] Pardoll DM. The blockade of immune checkpoints in cancer immunotherapy. Nat Rev Cancer. (2012) 12:252–64. doi: 10.1038/nrc3239 PMC4856023 22437870

[85] Topalian SL Hodi FS Brahmer JR Gettinger SN Smith DC McDermott DF. Safety, activity, and immune correlates of anti-pd-1 antibody in cancer. N Engl J Med. (2012) 366:2443–54. doi: 10.1056/NEJMoa1200690 PMC3544539 22658127

[86] Brahmer JR Tykodi SS Chow LQ Hwu WJ Topalian SL Hwu P. Safety and activity of anti-pd-L1 antibody in patients with advanced cancer. N Engl J Med. (2012) 366:2455–65. doi: 10.1056/NEJMoa1200694 PMC3563263 22658128

[87] Filley AC Henriquez M Dey M. Recurrent glioma clinical trial, checkmate-143: the game is not over yet. Oncotarget. (2017) 8:91779–94. doi: 10.18632/oncotarget.21586 PMC5710964 29207684

[88] Reardon DA Brandes AA Omuro A Mulholland P Lim M Wick A. Effect of nivolumab vs bevacizumab in patients with recurrent glioblastoma: the checkmate 143 phase 3 randomized clinical trial. JAMA Oncol. (2020) 6:1003–10. doi: 10.1001/jamaoncol.2020.1024 PMC7243167 32437507

[89] Mathewson ND Ashenberg O Tirosh I Gritsch S Perez EM Marx S. Inhibitory cd161 receptor identified in glioma-infiltrating T cells by single-cell analysis. Cell. (2021) 184:1281–98 e26. doi: 10.1016/j.cell.2021.01.022 33592174 PMC7935772

[90] Vivier E Tomasello E Baratin M Walzer T Ugolini S. Functions of natural killer cells. Nat Immunol. (2008) 9:503–10. doi: 10.1038/ni1582 18425107

[91] Moretta L Biassoni R Bottino C Cantoni C Pende D Mingari MC. Human nk cells and their receptors. Microbes Infect. (2002) 4:1539–44. doi: 10.1016/s1286-4579(02)00037-0 12505526

[92] Lanier LL. Nk cell recognition. Annu Rev Immunol. (2005) 23:225–74. doi: 10.1146/annurev.immunol.23.021704.115526 15771571

[93] Moretta A Bottino C Vitale M Pende D Biassoni R Mingari MC. Receptors for hla class-I molecules in human natural killer cells. Annu Rev Immunol. (1996) 14:619–48. doi: 10.1146/annurev.immunol.14.1.619 8717527

[94] Mistry AR O’Callaghan CA. Regulation of ligands for the activating receptor nkg2d. Immunology. (2007) 121:439–47. doi: 10.1111/j.1365-2567.2007.02652.x PMC2265965 17614877

[95] Cooper MA Fehniger TA Turner SC Chen KS Ghaheri BA Ghayur T. Human natural killer cells: A unique innate immunoregulatory role for the cd56(Bright) subset. Blood. (2001) 97:3146–51. doi: 10.1182/blood.v97.10.3146 11342442

[96] Luetke-Eversloh M Cicek BB Siracusa F Thom JT Hamann A Frischbutter S. Nk cells gain higher ifn-gamma competence during terminal differentiation. Eur J Immunol. (2014) 44:2074–84. doi: 10.1002/eji.201344072 24752800

[97] Remark R Alifano M Cremer I Lupo A Dieu-Nosjean MC Riquet M. Characteristics and clinical impacts of the immune environments in colorectal and renal cell carcinoma lung metastases: influence of tumor origin. Clin Cancer Res. (2013) 19:4079–91. doi: 10.1158/1078-0432.CCR-12-3847 23785047

[98] Weller M Fontana A. The failure of current immunotherapy for Malignant glioma. Tumor-derived tgf-beta, T-cell apoptosis, and the immune privilege of the brain. Brain Res Brain Res Rev. (1995) 21:128–51. doi: 10.1016/0165-0173(95)00010-0 8866671

[99] Friese MA Wischhusen J Wick W Weiler M Eisele G Steinle A. Rna interference targeting transforming growth factor-beta enhances nkg2d-mediated antiglioma immune response, inhibits glioma cell migration and invasiveness, and abrogates tumorigenicity *in vivo* . Cancer Res. (2004) 64:7596–603. doi: 10.1158/0008-5472.CAN-04-1627 15492287

[100] Friese MA Platten M Lutz SZ Naumann U Aulwurm S Bischof F. Mica/nkg2d-mediated immunogene therapy of experimental gliomas. Cancer Res. (2003) 63:8996–9006. 14695218

[101] Frisbie L Buckanovich RJ Coffman L. Carcinoma-associated mesenchymal stem/stromal cells: architects of the pro-tumorigenic tumor microenvironment. Stem Cells. (2022) 40:705–15. doi: 10.1093/stmcls/sxac036 PMC9406606 35583414

[102] Shi Y Du L Lin L Wang Y. Tumour-associated mesenchymal stem/stromal cells: emerging therapeutic targets. Nat Rev Drug Discovery. (2017) 16:35–52. doi: 10.1038/nrd.2016.193 27811929

[103] Lim EJ Kim S Oh Y Suh Y Kaushik N Lee JH. Crosstalk between gbm cells and mesenchymal stemlike cells promotes the invasiveness of gbm through the C5a/P38/zeb1 axis. Neuro Oncol. (2020) 22:1452–62. doi: 10.1093/neuonc/noaa064 PMC7566528 32179921

[104] Hossain A Gumin J Gao F Figueroa J Shinojima N Takezaki T. Mesenchymal stem cells isolated from human gliomas increase proliferation and maintain stemness of glioma stem cells through the il-6/gp130/stat3 pathway. Stem Cells. (2015) 33:2400–15. doi: 10.1002/stem.2053 PMC4509942 25966666

[105] Kong BH Shin HD Kim SH Mok HS Shim JK Lee JH. Increased *in vivo* angiogenic effect of glioma stromal mesenchymal stem-like cells on glioma cancer stem cells from patients with glioblastoma. Int J Oncol. (2013) 42:1754–62. doi: 10.3892/ijo.2013.1856 23483121

[106] Nakhle J Khattar K Ozkan T Boughlita A Abba Moussa D Darlix A. Mitochondria transfer from mesenchymal stem cells confers chemoresistance to glioblastoma stem cells through metabolic rewiring. Cancer Res Commun. (2023) 3:1041–56. doi: 10.1158/2767-9764.CRC-23-0144 PMC10266428 37377608

[107] Peng Z Wu Y Wang J Gu S Wang Y Xue B. Development and validation of a glioma-associated mesenchymal stem cell-related gene prognostic index for predicting prognosis and guiding individualized therapy in glioma. Stem Cell Res Ther. (2023) 14:56. doi: 10.1186/s13287-023-03285-9 37005685 PMC10068170

[108] Dominici M Le Blanc K Mueller I Slaper-Cortenbach I Marini F Krause D. Minimal criteria for defining multipotent mesenchymal stromal cells. The international society for cellular therapy position statement. Cytotherapy. (2006) 8:315–7. doi: 10.1080/14653240600855905 16923606

[109] Bhowmick NA Neilson EG Moses HL. Stromal fibroblasts in cancer initiation and progression. Nature. (2004) 432:332–7. doi: 10.1038/nature03096 PMC3050735 15549095

[110] Clavreul A Etcheverry A Chassevent A Quillien V Avril T Jourdan ML. Isolation of a new cell population in the glioblastoma microenvironment. J Neurooncol. (2012) 106:493–504. doi: 10.1007/s11060-011-0701-7 21928115

[111] Trylcova J Busek P Smetana K Jr. Balaziova E Dvorankova B Mifkova A. Effect of cancer-associated fibroblasts on the migration of glioma cells *in vitro* . Tumour Biol. (2015) 36:5873–9. doi: 10.1007/s13277-015-3259-8 25712375

[112] Clavreul A Guette C Faguer R Tetaud C Boissard A Lemaire L. Glioblastoma-associated stromal cells (Gascs) from histologically normal surgical margins have a myofibroblast phenotype and angiogenic properties. J Pathol. (2014) 233:74–88. doi: 10.1002/path.4332 24481573

[113] Galbo PM Jr. Madsen AT Liu Y Peng M Wei Y Ciesielski MJ. Functional contribution and clinical implication of cancer-associated fibroblasts in glioblastoma. Clin Cancer Res. (2024) 30:865–76. doi: 10.1158/1078-0432.CCR-23-0493 PMC10922678 38060213

[114] Zhang X Sun X Guo C Li J Liang G. Cancer-associated fibroblast-associated gene igfbp2 promotes glioma progression through induction of M2 macrophage polarization. Am J Physiol Cell Physiol. (2024) 326:C252–C68. doi: 10.1152/ajpcell.00234.2023 37982173

[115] Wu L Liu Q Li G Shi W Peng W. A cancer-associated fibroblasts related risk score (Cafscore) helps to guide prognosis and personal treatment for glioblastoma. Discovery Oncol. (2024) 15:420. doi: 10.1007/s12672-024-01314-4 PMC11387281 39254749

[116] Jain S Rick JW Joshi RS Beniwal A Spatz J Gill S. Single-cell rna sequencing and spatial transcriptomics reveal cancer-associated fibroblasts in glioblastoma with protumoral effects. J Clin Invest. (2023) 133:1–18. doi: 10.1172/JCI147087 PMC9974099 36856115

[117] Yuan D Tao Y Chen G Shi T. Systematic expression analysis of ligand-receptor pairs reveals important cell-to-cell interactions inside glioma. Cell Commun Signal. (2019) 17:48. doi: 10.1186/s12964-019-0363-1 31118022 PMC6532229

[118] Zhu X Fang Y Chen Y Chen Y Hong W Wei W. Interaction of tumor-associated microglia/macrophages and cancer stem cells in glioma. Life Sci. (2023) 320:121558. doi: 10.1016/j.lfs.2023.121558 36889666

[119] Harwood DSL Pedersen V Bager NS Schmidt AY Stannius TO Areskeviciute A. Glioblastoma cells increase expression of notch signaling and synaptic genes within infiltrated brain tissue. Nat Commun. (2024) 15:7857. doi: 10.1038/s41467-024-52167-y 39251578 PMC11385527

[120] Abdelfattah N Kumar P Wang C Leu JS Flynn WF Gao R. Single-cell analysis of human glioma and immune cells identifies S100a4 as an immunotherapy target. Nat Commun. (2022) 13:767. doi: 10.1038/s41467-022-28372-y 35140215 PMC8828877

[121] Xing J Cai H Lin Z Zhao L Xu H Song Y. Examining the function of macrophage oxidative stress response and immune system in glioblastoma multiforme through analysis of single-cell transcriptomics. Front Immunol. (2023) 14:1288137. doi: 10.3389/fimmu.2023.1288137 38274828 PMC10808540

[122] Wang L Jung J Babikir H Shamardani K Jain S Feng X. A single-cell atlas of glioblastoma evolution under therapy reveals cell-intrinsic and cell-extrinsic therapeutic targets. Nat Cancer. (2022) 3:1534–52. doi: 10.1038/s43018-022-00475-x PMC9767870 36539501

[123] Shi Q Xue C Zeng Y Yuan X Chu Q Jiang S. Notch signaling pathway in cancer: from mechanistic insights to targeted therapies. Signal Transduction Targeted Ther. (2024) 9:128. doi: 10.1038/s41392-024-01828-x PMC11128457 38797752

[124] D’Amico M De Amicis F. Aberrant notch signaling in gliomas: A potential landscape of actionable converging targets for combination approach in therapies resistance. Cancer Drug Resist. (2022) 5:939–53. doi: 10.20517/cdr.2022.46 PMC9771760 36627893

[125] Cai L Liu Y Li Y Liu B Cao Y Yang W. Trim37 interacts with ezh2 to epigenetically suppress ptch1 and regulate stemness in glioma stem cells through sonic hedgehog pathway. J Neurooncol. (2024) 169:269–79. doi: 10.1007/s11060-024-04726-y 38884661

[126] Thorlacius-Ussing J Jensen C Nissen NI Cox TR Kalluri R Karsdal M. The collagen landscape in cancer: profiling collagens in tumors and in circulation reveals novel markers of cancer-associated fibroblast subtypes. J Pathol. (2024) 262:22–36. doi: 10.1002/path.6207 37728068

[127] Liu H Tang T. Mapk signaling pathway-based glioma subtypes, machine-learning risk model, and key hub proteins identification. Sci Rep. (2023) 13:19055. doi: 10.1038/s41598-023-45774-0 37925483 PMC10625624

[128] Herbert A. Contextual cell death in adaptive immunity: selecting a winning response. Front Immunol. (2019) 10:2898. doi: 10.3389/fimmu.2019.02898 31921159 PMC6930443

[129] Lane R Cilibrasi C Chen J Shah K Messuti E Mazarakis NK. Pdgf-R inhibition induces glioblastoma cell differentiation via dusp1/P38(Mapk) signalling. Oncogene. (2022) 41:2749–63. doi: 10.1038/s41388-022-02294-x PMC9076540 35393545

[130] Liu J Wang X Chen AT Gao X Himes BT Zhang H. Znf117 regulates glioblastoma stem cell differentiation towards oligodendroglial lineage. Nat Commun. (2022) 13:2196. doi: 10.1038/s41467-022-29884-3 35459228 PMC9033827

[131] Wang H Zhao P Zhang Y Chen Z Bao H Qian W. Neurod4 Converts Glioblastoma Cells into Neuron-Like Cells through the Slc7a11-Gsh-Gpx4 Antioxidant Axis. Cell Death Discovery. (2023) 9:297. doi: 10.1038/s41420-023-01595-8 37582760 PMC10427652

[132] Korsos V Miller WH Jr. How retinoic acid and arsenic transformed acute promyelocytic leukemia therapy. J Mol Endocrinol. (2022) 69:T69–83. doi: 10.1530/JME-22-0141 36112505

