# Supplementary figures and images for: Molecular biology of the deadliest cancer – glioblastoma: what do we know?
Source: Front Immunol. 2025 Mar 21;16:1530305. doi: 10.3389/fimmu.2025.1530305 (PMC11968700; doi:10.3389/fimmu.2025.1530305)

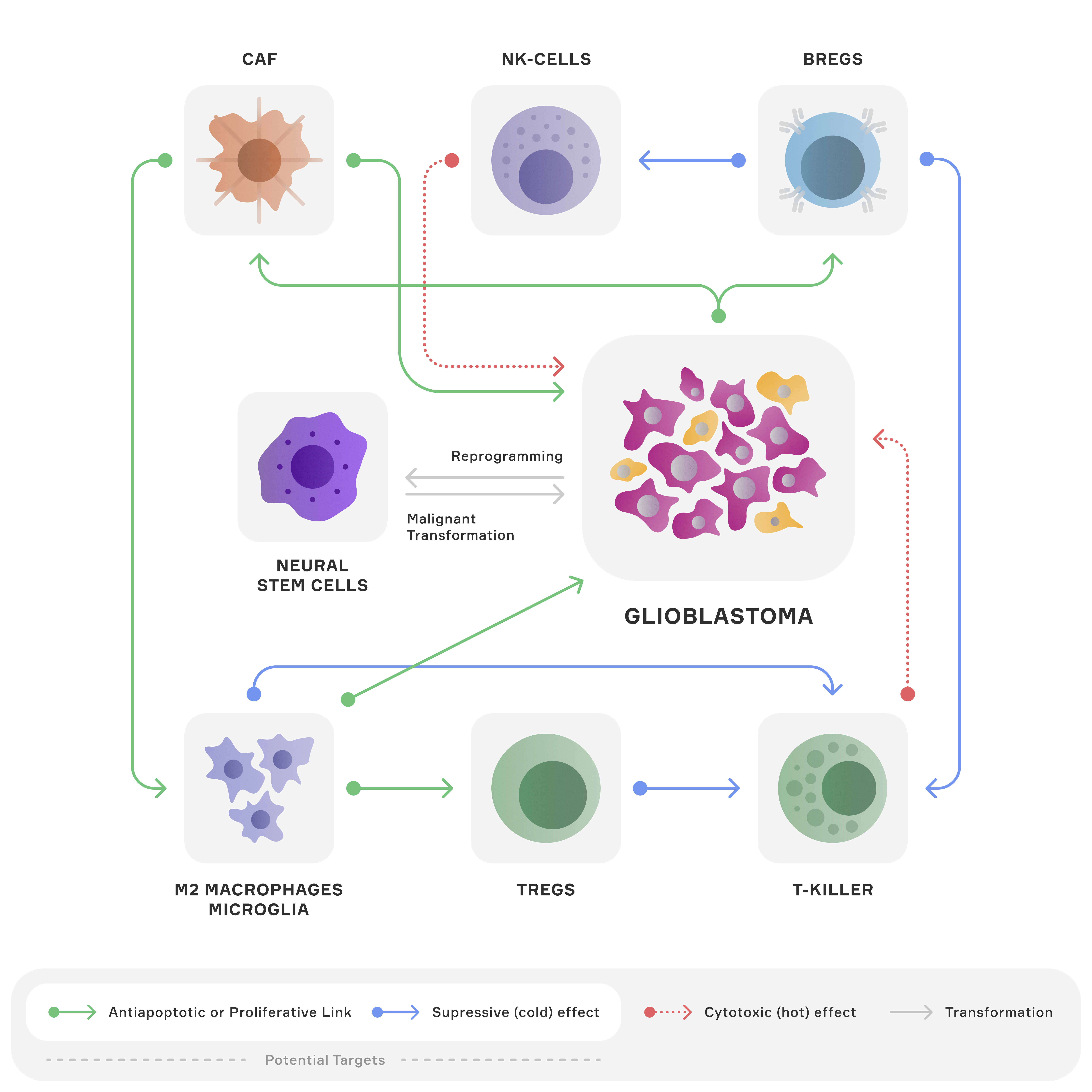

Supplement: Supplementary file 1 [file Image1.png]
